# Diagnostic biomolecules and combination therapy for pre-eclampsia

**DOI:** 10.1186/s12958-022-01003-3

**Published:** 2022-09-06

**Authors:** Jingqi Qi, Bingbing Wu, Xiuying Chen, Wei Wei, Xudong Yao

**Affiliations:** 1 International Institutes of Medicine, The Fourth Affiliated Hospital, Zhejiang University School of Medicine, No. N1, Shangcheng Avenue, Yiwu, 322000 China; 2 Zhejiang University-University of Edinburgh Institute (ZJU-UoE Institute), Zhejiang University School of Medicine, International Campus, Zhejiang University, 718 East Haizhou Road, Haining, 314400 China

**Keywords:** Preeclampsia, Diagnosis, Biomarkers, Therapeutics

## Abstract

Pre-eclampsia (PE), associated with placental malperfusion, is the primary reason for maternal and perinatal mortality and morbidity that can cause vascular endothelial injury and multi-organ injury. Despite considerable research efforts, no pharmaceutical has been shown to stop disease progression. If women precisely diagnosed with PE can achieve treatment at early gestation, the maternal and fetal outcomes can be maximally optimized by expectant management. Current diagnostic approaches applying maternal characteristics or biophysical markers, including blood test, urine analysis and biophysical profile, possess limitations in the precise diagnosis of PE. Biochemical factor research associated with PE development has generated ambitious diagnostic targets based on PE pathogenesis and dissecting molecular phenotypes. This review focuses on current developments in biochemical prediction of PE and the corresponding interventions to ameliorate disease progression, aiming to provide references for clinical diagnoses and treatments.

## Background

**Fig. 1:**
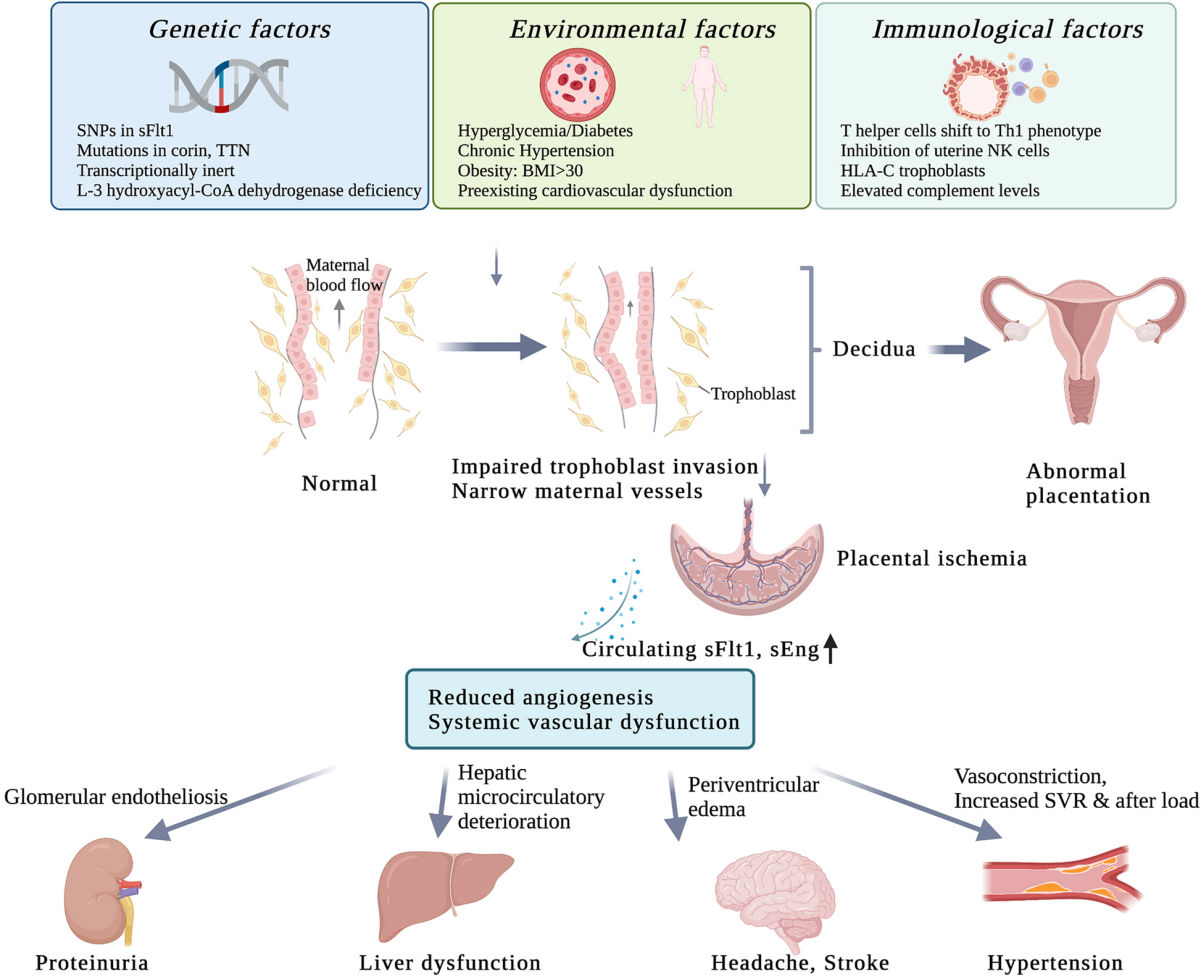
Pathophysiology of preeclampsia. Genetic factors, environmental factors and immunological factors are contributing factors to preeclampsia (PE). Genetic factors mainly include single nucleotide polymorphisms in sFlt1, mutations in corin and Titin genes, transcriptionally inert and L-3 hydroxyacyl-CoA dehydrogenase deficiency. Also, maternal hyperglycemia, diabetes, chronic hypertension, obesity and preexisting cardiovascular system may also induce the PE. Immunological factors mainly include the shift from T helper cells to Th1 phenotype, inhibition of uterine NK cells, increased HLA-C trophoblasts and elevated complement levels. These factors can lead to impaired trophoblast invasion and maternal vessel narrowing, causing placental ischemia and abnormal placentation. Then, the levels of circulating soluble fms-like tyrosine kinase 1 (sFlt1) and soluble endoglin (sEng) will increase, that reduces angiogenesis and causes systemic vascular dysfunctions such as proteinuria, liver dysfunction, headache, stroke and hypertension. (Abbreviation: BMI, body mass index; sFlt1, soluble fms-like tyrosine kinase 1; sEng, soluble endoglin; SNP, single nucleotide polymorphism; TTN, Titin)

Pre-eclampsia (PE) is a hypertensive complication during pregnancy that occurs at a rate of 3–5% worldwide [1], which has been subdivided into early-onset forms less than 34 weeks of gestation and late-onset forms of more than 34 weeks of gestation [2]. The pathophysiology of PE is still unclear. Increasing scientific evidence have suggested a series of stages of disease development (Fig. 1) [3–6]. Firstly, under the influence of genetic factors, environmental factors, and immunological factors, the placental insufficiency originates during the first and second trimesters of placentation [3, 7]. The genetic factors mainly include the imbalance of maternal and fetal soluble fms-like tyrosine kinase 1 (sFlt1) single nucleotide polymorphisms, decidual transcriptome, and heme oxygenase isoform [8]. Meanwhile, diabetes mellitus, hyperglycemia, and chronic hypertension in pregnancy can greatly raise the morbidity of PE [9]. Additionally, immunological factors such as placental T helper 1 cell predominance, decidual natural killer cells and immunogenic human leukocyte antigen-C on trophoblasts will also affect the PE progress. Then, during the late second and third trimesters, the invasive trophoblast superficial invasion narrows maternal vessels, leading to placental ischemia [8]. The oxidative stress and persistent hypoxia of placenta will increase the levels of sFlt1 and soluble endoglin (sEng), syncytial decries, and pro-inflammatory cytokines in the maternal circulation [10]. High levels of sFlt1 and sEng can result in systemic vascular dysfunction including proteinuria, hypertension, eclampsia and HELLP syndrome (hemolysis, elevated liver enzymes and low platelets). Accordingly, PE patients have a larger probability to deliver small-for-gestational age infants.

Appropriate antenatal care, management and treatment are promising to reduce the risk of PE with early identification. Currently, the diagnosis of PE is fundamentally based on the maternal characteristics including age, origin, body mass index (BMI), PE history, chronic hypertension, and conception method, followed by extrapolating and incorporating these characteristics into a designed mathematical formula to calculate the potential risk of PE occurrence [11]. By this traditional way, only 40% of all PE at false-positive rate (FPR) of 10% can be identified [12]. Besides, blood pressure (BP) is a routine test in antenatal care for PE assessment, and an exceeding threshold of 140/90 mmHg has been recognized as hypertension. Since the considerable variability among each individual hinders an accurate assessment of BP, the PE detection rates using BP ranges from 8 to 93% [13]. Based on these, the mean arterial pressure (MAP), showing higher predictability than systolic or diastolic BP, has been highly recommended by validated automated devices [14]. Patients who develop PE demonstrate the increase in MAP at 11–13 weeks of gestation, but the detection rate of using MAP in isolation was only 30% for early-onset PE at a 10% FPR, and the combined MAP with maternal characteristics only raised the rate to 62.5% [13]. To sum up, novel biomarkers and diagnosis techniques are urgently needed to improve the efficiency of current routines for PE diagnosis.

Maternal uterine artery blood flow is essential for the intrauterine environment, placental function and fetal growth. During the development of PE, abnormal placental perfusion leads to trophoblast damage, primary trophoblast invasion defects, and ultimately inadequate transformation of the maternal uterine vascular system [15]. The blood flow in the maternal blood vessels can be monitored by the uterine artery Doppler ultrasound and recorded by pulsatility index (PI). Increased uterine artery PI induced by poor placental perfusion is a typical sign of PE development [16]. The detection rate of using PI in isolation is approximately 77.3% for early-onset PE and 26.8% for late-onset PE at a 10% FPR, while the combination of PI with maternal characteristics enhances the detection rate of early-onset PE to 100% with minor improvement to 46.5% for late-onset PE at the same FPR [17]. If PI and MAP are used synergistically with mathematically calculating PE risks using maternal characteristics, it will exhibit a high detection rate of 89% for early-onset PE, but still only 57% for late-onset PE at a FPR of 10% [18]. Since PE-induced pulmonary embolism commonly causes increased levels of mean platelet volume (MPV) and red cell distribution width (RDW), the blood tests detecting platelet count, mean platelet volume, and red cell distribution width have been raised as significant markers of PE [19, 20]. Although hemo-diagnosis possesses several advantages of fast and low-cost, their specificity and sensitivity are still unsatisfactory. That is mainly because multiple interferences in plasma may make accurate detection difficult, including the effects of detection time, physiologic and metabolic changes on the blood components [21, 22]. As the major cases of the PE occur in late-onset forms, alternative biomarkers remain a pressing need.

Current treatments for PE mainly include the prenatal aspirin for women at high risk, betamethasone for PE patients in early stage, intravenous magnesium sulfate and real-time postpartum BP monitoring, though timely delivery of the fetus remains the only ultimate treatment [23]. Currently, the American College of Obstetrics and Gynecology does not recommend medication for mild to moderate hypertension in PE, because it cannot reduce the risk of disease progression and may increase the risk of fetal restriction [24]. Treatment of PE patients with severe hypertension requires pharmacological therapy such as labetalol, nifedipine and methyldopa [24]. However, recent evidence from animal studies suggests that amlodipine may be superior to nifedipine due to its induction of Arrb1 and subsequent downregulation of the AT1-B2 receptor complex, but more clinical evidence is needed [25]. Although there exists some challenges in current management of PE, innovative medical therapies are emerging. In this review, we discuss promising PE diagnostic tools by focusing on the links among pathways associated with PE pathogenesis and propose the applications of cutting-edge findings on impeding PE development.

## Methods

We studied the prediction, diagnosis and treatment of preeclampsia. A systematic search of PUBMED was performed to identify relevant studies by using the keywords of diagnosis or biomarkers or therapeutics of preeclampsia. Although we strived to include the current evidence on the topic, this is not a systemic review of the literature.

## Novel biochemical markers with PE occurrence

**Fig. 2:**
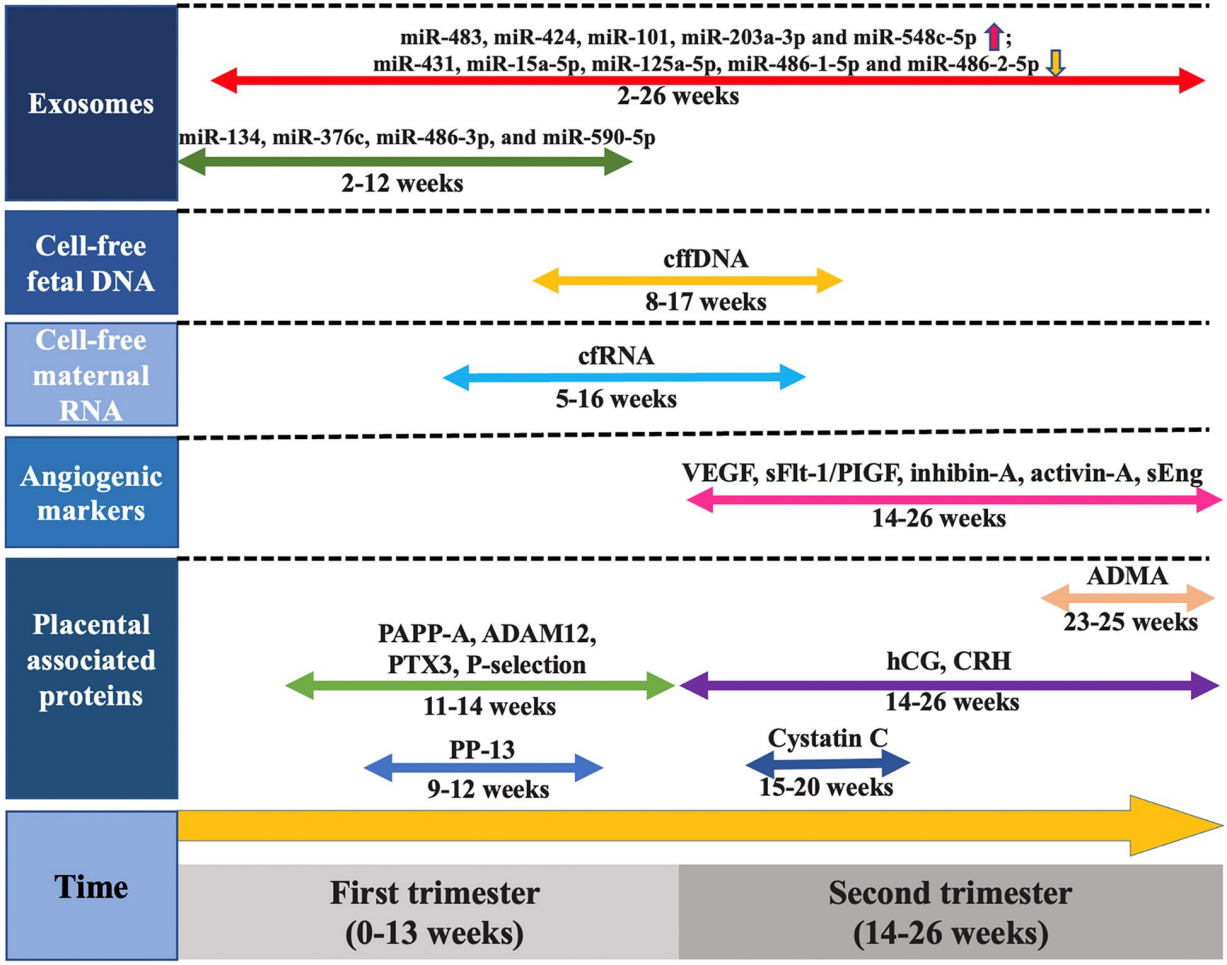
Current biochemical markers for early prediction of PE. A series of biochemical markers has been identified to predict the PE in the first and second trimesters, including exosomes, cell-free fetal DNA, cell-free maternal RNA, angiogenic markers and placental associated proteins. These markers possess specific detection time during the stages of pregnancy. (Abbreviations: ADMA, asymmetric dimethyl arginine; ADAM12, A disintegrin and metalloprotease12; CRH, corticotropin-releasing hormone; hCG, human chorionic gonadotropin; PTX, Pentraxin; PIGF, placental growth factor; PP-13, placental protein-13; PAPP-A, pregnancy-associated plasma protein A; sEng, soluble endoglin; sFlt-1, soluble fms-like tyrosine kinase 1; VEGF, vascular endothelial growth factor; cffDNA, cell-free fetal DNA; miR, microRNA.)

To date, continuous understanding of pathogenic mechanisms, especially those underlying molecule changes in maternal blood, has inspired various targeted biochemical markers to identify PE disease process. Figure 2 shows promising predictive markers with their unique functions during PE pregnancy.

### Placental associated proteins

Proteases play key roles in the placental remodeling process. The secretion of placental protein 13 (PP13), pregnancy-associated placental protein A (PAPP-A) and a disintegrin and metalloprotease 12 (ADAM12) positively correlates with the size of the placenta [26]. Therefore, reduction in these proteins can be recognized as the first trimester biomarkers of PE. PP13 from the galectin family produced by the syncytiotrophoblast of fetomaternal interference is reduced in PE patients even at 9–12 weeks of gestation [27]. The use of PP13 with PI leads to 90% detection rate in PE [28], and the combination of PP13 and PAPP-A reaches 91% of sensitivity and specificity at the first and second trimesters of PE [29]. Moreover, a significant decline in ADAM12 and PAPP-A levels at 11–14 weeks of gestation has been observed in PE patients compared with control group. In contrast, another study claimed that ADAM12 and PAPP-A in combination with maternal characteristics only identified 50% and 48% of patients who developed PE at a fixed FPR of 10%, respectively [30]. The prediction of PE has been analyzed by logistic regression using ADAM12 multiples of median (MoM), PAPP-A MoM, and uterine Artery Doppler pulse index MoM. To compare the screening efficiency of the nonparametric U statistic model, the sensitivity, specificity, and area under the subject operating characteristic curves were measured. Accordingly, combining the early pregnancy parameters or even the first-trimester parameters with PI cannot improve the predictive efficiency of the model (50%) [31].

Various circulating hormones, including human chorionic gonadotropin (hCG) [32, 33] and corticotropin-releasing hormone (CRH) [34], have been reported to elevate in PE cases at the second trimester, indicating their prognostic prediction capability in the late-onset PE. Nevertheless, no further clinical trial within diverse populations has proven their high sensitivity in PE detection. Moreover, the endothelial dysfunction is a key mediator during PE [35]. First, the placental selectin involved in leucocyte trafficking changes in PE progression. Increased E-and L-selectin but decreased P-selectin have been detected in the early-onset of PE [36]. Pentraxin 3 (PTX3), an inflammation biomarker, has been observed to elevate in the first trimester at 11–14 weeks in patients who subsequently developed PE [37, 38]. Similarly, asymmetric dimethylarginine (ADMA) also found to be increased in the first trimester at developing early-onset PE [39]. PTX3 and ADMA might possess mechanistic links between vascular effects in PE and later cardiovascular risk [40], and the abnormal elevations in both were able to segregate high risk PE patients at early stage [41].

Furthermore, renal blood flow and glomerular filtration rate (GFR) were commonly observed to be decreased in PE patients [42]. Proteinuria will be raised responding to the injury of the glomerular capillary. Currently, the gold standard for PE diagnosis is the 24-h urine collection that assesses proteinuria. Moreover, the 12-h urine collection for proteinuria also shows high clinical efficiency (sensitivity, 92%; specificity, 99%) with convenient management [43]. However, the results may be unreliable, because the inaccurate collection may affect the results of PE diagnosis. The actual prevalence of significant proteinuria is varied by laboratory processing methods [44]. To combat this, more convenient and accurate diagnostic tests that are capable of prognosticating kidney injury of PE patients have been developed recently. Valdes et al. [45] reported that the protein/creatinine ratio (PCR) yielded a high positive predictive value of 96.4% at FPR 4.4% (AUC, 08,802) which could substitute the 24-h urine collection. Unfortunately, creatinine is not sensitive to the real GFR decline, as it could be largely affected by the secretion from renal tubules, muscle mass, protein uptake, and physical activity [46]. Instead, Cystatin C filtered by glomerulus provides potential advantages for accurate PE prediction. Patients with high Cystatin C concentration have been widely reported to have a risk for subsequent PE development [47–49]. To conclude, more accurate studies focusing on the efficiency of placental associated protease are needed.

### Angiogenic markers

Angiogenesis in the placental vascularization refers to the remodeling of new blood capillaries from the pre-existing vasculature mediated by the synergistic actions of pro- and anti-angiogenic factors [50]. Both endothelial cell proliferation and migration are activated by vascular endothelial growth factor (VEGF) and placental growth factor (PIGF) that restore the vascular integrity [51]. Soluble Feline McDonough Sarcoma-like tyrosine kinase 1 (sFlt-1), a decoy receptor, sequesters VEGF and PlGF interaction with the VEGF-1 receptors on endothelial cells to impair the endothelial cell–cell communication and cause vascular dysfunction and hypertension [52]. As a result, PE patients normally show increased circulating sFlt1 concentrations but decreased levels of VEGF and PlGF. Single PIGF determination has been reported to predict PE with a sensitivity of 100% at a concentration threshold of 120.16 pg/mL and a diagnosis accuracy of 70.8%, while sFlt-1 only exhibited the diagnostic accuracy of 76.9%, sensitivity of 73.1% and specificity of 80.8% [53]. Hence, Zeisler et al. [54] suggests that a sFlt-1/PIGF ratio of less than 38 can discover the short-term absence of PE in women with suspicion of PE. A recent study demonstrated that PE diagnostic performance using the sFlt-1/PIGF ratio can achieve 95% sensitivity and 91% specificity which is superior to VEGF, PIGF or sFLt-1 [55]. Lately, several studies have attempted to use the VEGF genetic polymorphisms for early prediction of PE but there remains a lack of clinical competence [56, 57].

Chronic placental hypoxia exposure to the pro-inflammatory cytokines gradually increases the secretion of transforming growth factor-β (TGF-β) superfamily including inhibin-A, activin-A and soluble endoglin (sEng), which can be considered as endocrine markers for disorder prediction [58]. However, sEng-based diagnostic accuracy in maternal serum was only shown with 62.1% sensitivity and 56.8% specificity [53]. Even the combinations of sEng and PP13, PAPP-A, ADAM12, inhibin A or activin A only achieved 60%-80% sensitivity and approximately 80% specificity in the first or early second trimester [59], which causes poorer results than that of the sFlt-1/PIGF.

Overall, the angiogenic markers may be useful as a screening test, but no solid evidence performs to date documenting clinical implementation. The clinical studies are limited by single national region, small scale of participant population and lacking convenience sampling.

### Cell-free fetal DNA

Maternal blood containing the placenta-derived cell-free fetal DNA (cffDNA) have provided a new way for noninvasive prenatal testing (Table 1). CffDNA plasma level increases during PE pregnancy at an early gestational age and can reflect the placental apoptosis and necrosis [60]. The principle of cffDNA analysis is based on the promoter, RASSF1A gene (Ras association domain family 1 isoform A), which was hypermethylated in placenta but hypomethylated in maternal blood cells [61, 62]. Saraswathy et al. [63] observed hypermethylated RASSF1A concentrations elevated 3.3-fold in PE before the occurrence of clinical symptoms. At gestational age of 8–17 weeks, the cffDNA concentration turned positive before the PE onset, with 100% sensitivity and 50% specificity [64]. This is consistent with a recent study, in which maternal blood at 11–14 weeks of pregnancy estimating a cut-off value of cffDNA concentration at 22.54 GE/ml could predict the PE with 85.0% sensitivity and 81.8% specificity [65]. Except for RASSF1A, other gene makers have been demonstrated to identify the cffDNA. For instance, the Sex-degerming region Y [66–68], β-globin [68] and DYS [60] have shown great potential for PE prediction. As shown in Table 1, the quantity of cffDNA is a promising marker [69]. Moreover, further studies are necessary to minimize the heterogeneity of population scale, result assessments and evaluation methods.

**Table 1:** Application of cffDNA associated genes in PE prediction

Marker gene to quantify	Gestational age (in weeks)	Results in PE prediction
Hypermethylated RASSF1A	8–17	cffDNA concentration at cutoff value of 7.49 GE/ml showing 100% sensitivity and 50% specificity [60]
	11–13	cffDNA concentration at cutoff value of 512 GE/ml showing 100% sensitivity and 50% specificity [60]
	15–28	cffDNA concentration increased 3.3-fold [59]
	21- 40	The positive correlations of hypermethylated RASSF1A with PAPP-A, PP-13 and urine protein in PE [59]
DSCR3, RASSF1A, HYP2	6–14	The combination of DSCR3, RASSF1A, HYP2 and PAPP-A with 66.9% of detection rate at 10% of FPR [58]
Y chromosome specific gene	15–20	cffDNA concentration at cutoff value of 2.62 GE/ml showing 90% sensitivity and 85% specificity for early-onset PE [64]

### Cell-free RNA

Plasma cell-free RNA (cfRNA), a non-invasive manner, possesses a marked and stable level in the early gestation of PE patients (Table 2). Thus, cfRNA can reveal the pregnancy progression and determine the PE risk months before clinical symptoms present. The changes of cfRNA are enriched for genes specific to the neuromuscular, endothelial and immune cells and tissues, reflecting the correlation between PE progression and maternal organ health [70–73]. According to Mira et al., the identification and independent validation of a panel of 18 genes measured between 5 and 16 weeks of gestation can form the basis of a liquid biopsy test, shown in the Table 2 [74]. The cfRNA extracted from a single blood draw can track the pregnancy progression at the placental, maternal and fetal levels and reliably predict PE with a sensitivity of 75% and a positive predictive value of 32.3% [75]. Notably, the cfRNA characteristics are independent of various clinical factors including the maternal age, body mass index, and race, cumulatively accounting for less than 1% of the model variance. cfRNA also provides molecular evidence for the pathogenesis of PE caused by early placental abnormalities and systemic endothelial dysfunction [75]. Regardless of the type or severity of onset, placental signaling in PE is weakened, and the platelets and endothelial cells drive changes in cfRNA in PE patients, especially before the 20-week gestation. The increase in cell type-specific cfRNA may occur in part through the cellular signal transduction and secretion. Congenital and adaptive immune systems are also responsible for changes in cfRNA associated with significant metastases in the bone marrow, T cells, B cells, granulocytes, and neutrophils, consistent with previous studies on maternal placental interfaces and PE [76–78]. Generally, cfRNA has been considered as a novel, efficient and non-invasive method to predict PE in early stage and discover the underlying signaling pathways of PE pathology.

**Table 2:** Preeclampsia prediction relies on 8 increased cfRNA genes and 10 decreased cfRNA genes (Summarized from Moufarrej et al., 2022 [70])

Gene trends	Marker gene to quantify	Biological process	Molecular function
Enriched in PE	DERA (Deoxyribose-phosphate aldolase)	- Pentose-phophate shunt - Deoxyribose phosphate catabolic process	- Deoxyribose-phosphate aldolase activity - Protein binding
	KIAA 1109	- Synaptic vesicle endocytosis - Regulation of epithelial cell differentiation - Endosomal transport	- Protein binding
	NMRK1 (Nicotinamide riboside kinase 1)	- NAD metabolic process - Phosphorylation - NAD biosysnthetic process	- Ribosylnicotinamide kinase activity
	PI4KA (Phosphatidylinositol 4 kinase alpha)	- Multi-organism membrane organization - Phosphorylation - Viral replication complex formation and maintenance	- Kinase activity - Phosphatidylinasitol kinase activity - Protein binding
	PRTFDC1 (Phosphoribosyl transferase domain containing 1)	- Guanine salvage - GMP catabolic process - Purine ribonucleoside salvage	- Protein homodimerization activity - Hypoxanthines phosphoribosyltransferase activity
	Y_RNA	NR	NR
	Y_RNA	NR	NR
	YWHAQP5 (YWHAQ pseudogene 5)	NR	NR
Decreased in PE	CAMK2G (Calcium/ calmodulin dependent protein kinase II gamma)	- Nervous system development - Protein phopharylation - Regulation of neuron projective development - Regulation of skeletal muscle adaptation	- Calcium-dependent protein serine/ threonine phosphatase activity - Identical protein binding - Protein homodimerization activity
	FAM46A (Terminal mucleotidyltransferase 5A)	- mRNA stabilization - Response to bacterium	- RNA adenynltransferase activity - Protein binding - RNA binding
	LRRC58 (Leucine rich repeat containing 58)	NR	NR
	MYLIP (Myosin regulatory light chain interacting protein)	- Nervous system development - Regulation of low-density lipoprotein particle receptor catabolic process	- Ubiquitin protein ligaseactivity - Cytoskeletal protein binding - Protein binding - Metal ion binding
	NDUFV3 (NADH: ubiquinone oxidoreductase subunit V3)	- Mitochondrial electron transport - NADH to ubiquinone	- NADH dehydrogenase activity - Protein binding - RNA binding
	PYGO2 (Pygopus family PHD finger 2)	- Positive regulation of chromatin binding - Developemental growth - Regulation of histone H3-K4 methylation - Mammary gland development	- Chromatin binding - Protein binding - Metal ion binding - Histone acetyltransferase regulator activity - Histone binding
	RNF149 (Ring finger protein 149)	- Ubiquitin-dependent protein catabolic process - Regulation of protein stability - Protein ubiquitination	- Ubiquitin protein ligase activity - Metal ion binding
	TFIP11 (Tuftelin interacting protein 11)	- Biomineral tissue development - Negative regulation of protein binding - Spliceosomal complex disassembly	- Protein binding - Nucleic acid binding
	TRIM21 (Tripartite motif containing 21)	- Negative regulation of protein deubiquitination - Regulation of protein localization - Regulation of gene expression	- Zinc ion binding - Transcription coactivator activity - Identical protein binding
	USB1 (U6 snRNA biogenesis phosphodiesterase 1)	- U6 snRNA 3’-end processing - RNA splicing	- Poly (U)-specific exoribonuclease activity

*Abbreviation NR* Not reported

### Exosomes

Exosomes secreted by placenta increase in the PE maternal circulation, and their abilities for PE pathophysiology evaluation and disease prediction have been investigated [79–81]. Plasmatic exosomes from PE patient deliver abundant sFlt-1 and sEng to endothelial cells, causing vascular dysfunctions [82]. As mentioned before, a candidate marker PlGF also presented in exosomes, achieving 100% sensitivity and 78.6% specificity of PE prediction [83]. As PE can disrupt the immune balance, the quantification of exosomal Th1/Th2 cytokines including increased IL-2 and TNF-α with decreased IL-10 shows clinical significance in PE diagnosis [84]. In addition to the effect on protein, the placental-derived exosomes can also alter the RNA cargo [85, 86]. A study mapping the microRNA profiles in plasma exosomes extracted from maternal plasma of PE patients compared with those from normal pregnancies showed significantly different expression levels of miR-134, miR-376c, miR-486-3p and miR-590-5p in the first trimester (2–12 weeks) [87]. Moreover, novel miR-483, miR-424, miR-101, miR-203a-3p and miR-548c-5p were reported to be downregulated [88–92], while miR-431, miR-15a-5p, miR-125a-5p, miR-486–1-5p and miR-486–2-5p was aberrantly upregulated in the exosomes of PE patients [93–96]. Based on these identifications, exosomal miRNAs as a critical factor significantly expands the prospects for PE prediction. New detection methods such as microarrays chip, molecular beacons, and electrochemical sensors show the sensitivity and specificity for evaluating exosomes with heterogeneous subtypes [97], but systemic high-throughput analysis of exosomal RNA is still challenging as it translates from bench to bedside.

### Single-cell transcriptomes

The single-cell RNA sequencing offer unique opportunity for identifying the abnormal gene expression with high resolution in tissues and organs including the trophoblast-decidual interactions [98]. The Illumina Hiseq and Genome Analyzer II systems are popular tools in the placental research and have been extensively used thanks to the low-cost. The first single-cell atlas of the maternal–fetal interface was reported in 2018, revealing the landscape of early pregnancy. An array of unique fetal cell types derived from the early embryo, including villous cytotrophoblast cells, syncytiotrophoblast cells, extravillous trophoblast cells and maternal immune cells, have been detected [98]. Taking advantage of this discovery, Guo et al. [99] further compared both placental and peripheral blood transcriptomes to distinguish the fetal and maternal differences between women diagnosed with early- and late-onset PE. Interestingly, classical biomarkers such as sFlt-1, sEng and PAPP-A were only upregulated in the early-onset PE while the maternal blood-derived factors EBI3, IGF2, ORMDL3, GATA2 and KIR2DL4 were identified as new biomarkers for the late-onset PE. In the future, increasing PE diagnostic studies using single-cell transcriptomes will undoubtedly help to drive the field forward.

### Diagnostic recommendation

As shown above, while individual biomarkers demonstrate the connections between PE progression and secreted factor imbalance, none of them have demonstrated sensitivity and to be used as a test in isolation. Multiparametric biomarker testing may offer a path to improve the PE diagnosis. Early-onset PE appears less frequently (0.4–1%), but causes more significant disease burden due to prematurity, fetal growth restriction, and increased long-term maternal cardiovascular morbidity, while late-onset PE is regarded as secondary to maternal cardiovascular and metabolic predisposition for endothelial dysfunction [100]. In this case, splitting PE screening into two arms is recommended, including an early integrated test at 11–13 weeks and a second screen at 30–33 weeks for evaluating cases with late-onset PE [101].

Placental proteins, cffDNA and exosomes exhibit positive correlations with PE development, but considerable heterogeneity exists in the diagnostic accuracy according to current research. Hence, for the screening of low-risk population, biochemical biomarkers and Doppler ultrasonography are not recommended [102]. Currently, several organizations suggest screening for early-onset PE by obtaining BP measurements incorporating the maternal characteristics and urinalysis for proteinuria at each antenatal visit as adequate [103]. Subsequently, the late-onset PE assessment would depend on the changes in angiogenic factors (PIGF/sFlt-1), indicating an ideal predictive rate for estimating the onset of PE post 34 weeks of gestation.

## Clinical management of PE

To avoid neonatal respiratory distress syndrome in fetus born with preterm birth, delivering the placenta is the only cure for PE. Besides, PE has long-term implications for women. For instance, the occurrence of high blood pressure within 2 years of birth in women with PE history is sixfold higher than in women with no history of PE [104]. Therefore, effective prevention and treatment for this condition are sorely needed. Firstly, PE women should practice good health habits in the antenatal period, including sleeping adequate amounts, consuming a high-protein diet, ceasing smoking, exercising mildly to moderately, as well as maintaining a healthy weight. Additionally, salt restriction and bed rest are not recommended in women at risk of PE, and folic acid together with multi-vitamins are preferred to prevent spina bifida and help normal brain development.

Though detailed expectant managements of PE in the international clinical practice guidelines are still inconsistent, their principles primary rely on two characteristics: the gestational age and the disease severity. We encourage a delivery for women with confirmed PE at 37 weeks of gestation. Elective delivery beyond 34 weeks should be considered. In cases of severe PE with IUGR below 26 weeks, the evidence suggests that termination of pregnancy might be a necessary form of management to prevent maternal morbidity [105, 106]. Regarding to maternal-neonatal outcomes, clinicians need to fully compare the risk of elective delivery against expectant management of severe PE less than 34 weeks of gestation to achieve optimal maternal and child outcomes. We do not encourage elective delivery for PE before 34 weeks of gestation unless failure to correct severe maternal hypertension or maternal complications (e.g. eclampsia, liver failure, kidney failure, disseminated intravascular coagulation, placental abruption, HELLP syndrome and acute pulmonary edema), non-reassuring fetal status or unavailable monitoring. Prior to 34 weeks of gestation, expectant management for severe PE patients should be the first priority at facilities with high volume maternal and neonatal intensive care resources.

Magnesium sulfate has been considered as the first-line therapy for the prophylaxis against maternal eclampsia. Magnesium sulfate has the effects on lowering maternal BP as well as fetal neuroprotection [107], demonstrating superiority to other anticonvulsants in the prevention of maternal eclampsia. The common dose protocol for magnesium sulfate in PE is a 4 to 6 g intravenous (IV) loading dose with a continuous 1 g/h IV maintenance dose. Mild side effects of magnesium sulfate mainly include fever, flushing, nausea, vomiting, muscle weakness, dizziness and irritation at the injection site [108]. In randomized trials, the reported rates of these side effects range from 15 to 67% [109–111]. In addition, serious side effects such as respiratory depression and postpartum bleeding require monitoring of the correct dose. Maternal deaths from the overdose of magnesium have been reported in previous clinical trials [112]. As for women with gestational hypertension (Systolic BP ≥ 140 mmHg and/or Diastolic BP ≥ 90 mmHg), antihypertensive agents are recommended to prevent maternal morbidity and fetus injury. For instance, Labetalol, a beta-blocker, is the first-line treatment for hypertension in pregnancy. Nifedipine, a calcium channel blocker, is an alternative if labetalol is not suitable.

As for severe PE patients at 26–34 weeks gestation, antenatal corticosteroid therapy for acceleration of fetal lung maturity is recommended. Glucocorticoids can trigger the synthesis of ribonucleic acid that codes for proteins involved in the phospholipid biosynthesis or glycogen breakdown [113]. Epidemiological evidence and animal studies suggest that the prenatal exposure to corticosteroids may lead to adverse long-term consequences [114]. Some animal studies have demonstrated impaired glucose tolerance and elevated blood pressure in adult animals following prenatal exposure to corticosteroids [115–117]. In addition, premature and full-term infants exposed to a single course of corticosteroids can cause reduced brain growth [118, 119]. Thus, the side effects of corticosteroid therapy will be monitored carefully.

During pregnancy, physical monitoring and assessments are required to detect the PE progression. The blood pressure, respiratory rate, and continuous cardiotocography are needed to be considered. Additionally, the maternal assessments mainly include urea and electrolytes, full blood count and platelets, liver function test/ lactate dehydrogenase, coagulation screen, group and hold serum.

## Novel PE treatments

**Fig. 3:**
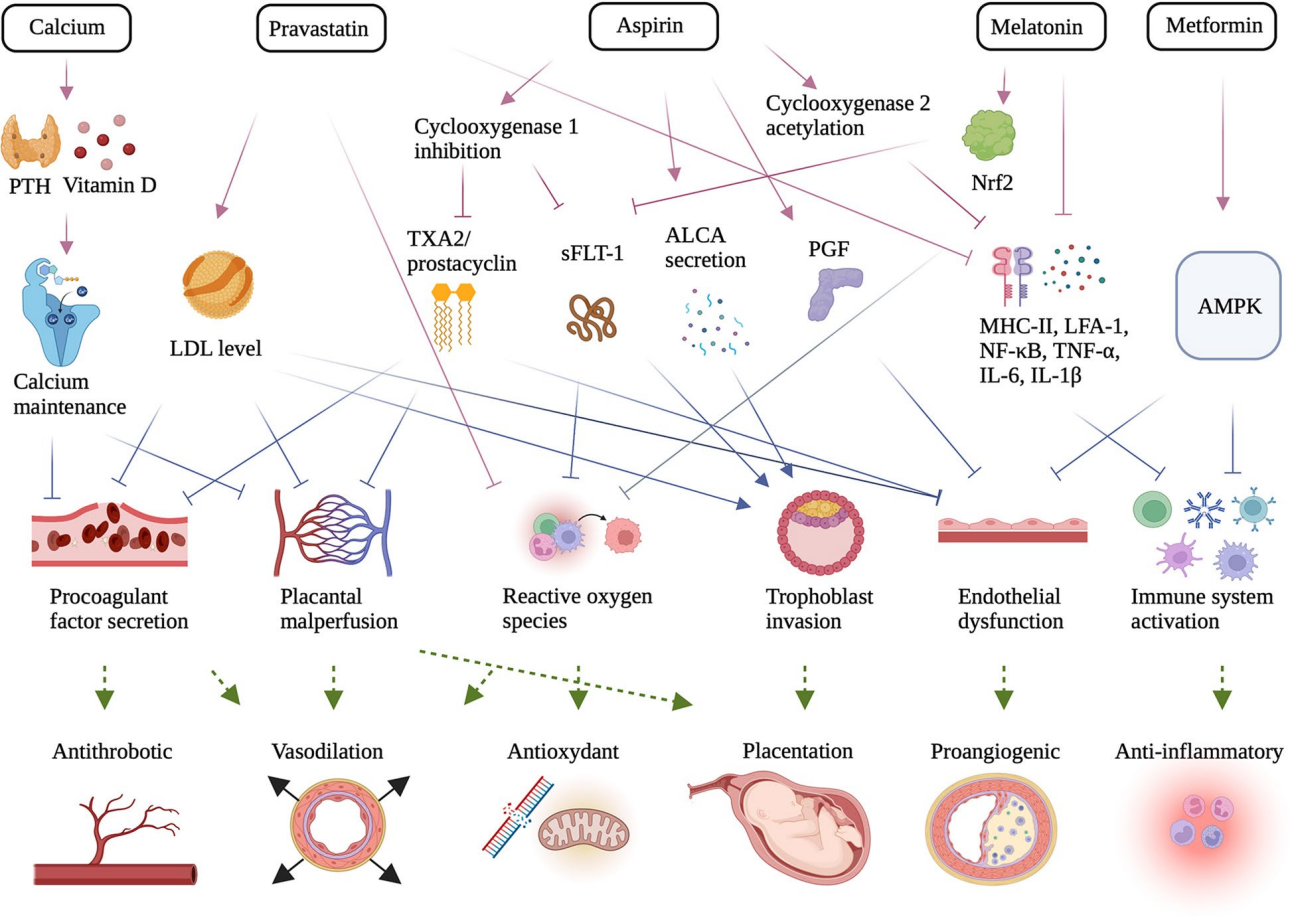
Novel managements of preeclampsia (PE). Increasing treatments of PE have been investigated, including Calcium supplement, Pravastatin, Aspirin, Melatonin and Metformin. Based on specific signaling pathways, the drugs target on the procoagulant factor secretion, placental perfusion, reactive oxygen species, trophoblast, endothelial function and immine system. Through improving the antithrombus, vasodilation, antioxidant, placentation, angiogenesis and inflammation, these drugs can finally alleviate the symtoms of PE. **(**Abbreviation: ALCA, activated leukocyte cell adhesion molecule; AMPK, 5’ adenosine monophosphate-activated protein kinase; IL-6, interleukin 6; IL-1β, interleukin-1β; LDL, low-density lipoprotein; LFA-1, β2-integrin; MHC-II, major histocompatibility complex II; NF-κB, nuclear factor kappa light chain enhancer of activated B cells; Nrf2, Nuclear factor erythroid 2-related factor 2; PGF, placenta growth factor; PTH, parathyroid hormone; sFLT-1, soluble fms-like tyrosin kinase-1; TXA2, Thromboxane A2; TNF-α, tumor necrosis factor-α)

PE, characterized by the release of anti-angiogenic factors and increased oxidative and inflammatory stress, jointly causes endothelial dysfunction, systemic maternal vascular disorder and hypertension [120]. Based on this, current treatments of PE targeting anti-angiogenic factors, inflammation, oxidative stress, endothelial injury and dysfunction have been researched (Fig. 3).

**Pravastatin** shows potential effects to prevent and treat PE through promoting the angiogenic signaling, endothelial colony-forming cell function and protein expression [120, 121]. They also play a role in reducing blood lipid, improving the vascular endothelial cell function, reducing the oxidative stress and inflammatory injury of systemic vascular systemic [122]. Though various animal experiments have achieved great progress of the application of pravastatin, its clinical use has been delayed, mainly because statins are classified by the food and drug administration (FDA) as class X [123, 124]. That means that the pravastatin may lead to fetal abnormalities.

In the past decade, pravastatin has shown its effect on preventing PE in two pregnancies, and it did not show teratogenic effects on pregnant women. In a cohort study of 890,000 people in the United States, approximately 1200 pregnant women who took statins in the first trimester of pregnancy experienced no increased incidence of congenital malformations [125]. Other organizations have reported similar epidemiological results [126, 127]. Although pravastatin has been used in a small number of high-risk pregnant women for several years, randomized controlled trials have not been reported. Recent studies have shown that pravastatin has favorable pharmacokinetic characteristics during pregnancy, and its safety has been confirmed by accumulating case studies [128]. In the future, it is vital to figure out the targets and duration of use. Additionally, large-scale clinical studies are needed to validate whether pravastatin may be an effective drug for PE prevention and treatment, improving outcomes for pregnant women and newborns at risk for PE.

**Metformin** has been reported to reduce the secretion of sFlt-1 from placenta as one of the markers of endothelial dysfunction. It can promote angiogenesis through inhibiting the mitochondrial electron transport chain, thus implying its potential to treat PE [129]. Metformin, with a molecular weight of 129 Dalton, can be directly diffused across the placenta without affecting the transfer of glucose. Double infusion of human placental lobules in vitro showed a lag time of rapid transfer of metformin from maternal to fetal circulation of 1.7 $\pm$ 0.28 min. During a randomized controlled clinical trial, nondiabetic or obese pregnant women were randomly assigned to receive metformin at 12–16 weeks of gestation [130]. The starting dose of 500 mg/day was increased to a maximum tolerable dose not exceeding 2500 mg. Accordingly, metformin decreased the frequency of PE, which is consistent with the results of a previous Meta-analysis [131].

Additionally, metformin had no significant effect on the birthweight, maternal weight, or combined adverse pregnancy outcomes. An in vivo study reported that metformin was detected in the neonatal cord blood of women with polycystic ovary syndrome treated with metformin during pregnancy, while such concentration of metformin in fetal umbilical arteries and umbilical veins are negligible [132]. Several meta-analyses have demonstrated that metformin has no effect on embryonic development including congenital malformations, and metformin is currently classified as class B in the United States and class C in Australia [133–135]. In pregnant women, adverse effects have been reported mainly in the gastrointestinal tract including nausea and diarrhea [135]. In addition, long-term use may produce some side effects, such as mild erythema and reduced absorption of vitamin B12 [136]. Therefore, although metformin can reduce the incidence of PE, the dose and duration of use need to be properly controlled on a case-by-case basis.

**Aspirin** has been initially used for PE prevention via the inhibition of platelet aggregation. Patients with high PE risk should be treated with antiplatelet therapy, regardless of gestation stage [137]. Aspirin acts on a variety of immune cells and exert anti-inflammatory and immunomodulatory effects. First of all, Aspirin inhibits the natural killer cell activity induced by melanoma [138], and inhibits the secretion of macrophage derived cytokines including lipopolysaccharide induced NF-κB and TNF-α [139]. Aspirin also regulates the maturation and differentiation of dendritic cells, thus affecting their functions such as activating downstream T cell proliferation [140]. Besides, Aspirin increases the number of Treg, and indirectly enhances the function of Treg by inducing the decrease of costimulatory factor expression on immune tolerant dendritic cells and the upregulation of costimulatory factor expression required for Treg activation [140, 141]. The use of Aspirin reduces the risk of proteinuric PE by 18%, preterm birth less than 37 weeks by 9%, fetal deaths, neonatal deaths or death before hospital discharge by 14% as well as small gestational age and serious adverse pregnancy outcomes [142]. Most clinical practice guidelines for pregnancy hypertension recommend a low-dose Aspirin therapy for the prevention of PE in high-risk women [143]. Currently, four randomized clinical trials have shown that low-dose Aspirin can significantly minimize the incidence of PE [144, 145]. The most recent trial applying 1776 subjects found that Aspirin apparently reduced the incidence of PE from 11 or 14 weeks to 36 weeks of pregnancy, suggesting an early supplement from 3 months or 6 months of pregnancy. Evidence also showed that Aspirin prophylaxis significantly reduces the early PE onset as well as preterm birth [146]. In our center, a daily dosage of 50–150 mg Aspirin taken at bedtime is highly recommended to patients with a PE history, initiating from 12 or 16 weeks until 35 weeks of gestation. To sum up, systematic studies on its regulatory effect on maternal fetal immunity in PE will be continued.

**Calcium** supplementation (≥ 1 g/day) during pregnancy may significantly decrease the risk of PE and preterm birth, especially in patients with a calcium diet deficiency (< 600 mg/d) [147]. Furthermore, its efficiency is highly correlated with the time of supplementation, as only taking calcium at early pregnancy stage may play a role in decreasing PE occurrence and pregnancy loss [148]. Recently, a systemic Meta-analysis of 27 randomized trials involving 28 492 pregnant women showed that high doses (1.2 to 2 g/ day), medium doses (0.6 to 1.2 g/ day) and low doses (< 0.6 g/ day) of calcium supplementation were associated with a reduced risk of PE [149]. However, further studies of direct concentration comparisons are needed to determine the ideal calcium dose for PE prevention.

**Melatonin** is an endogenous antioxidant that can improve PE maternal condition. In primary trophoblast cells, melatonin increases the release of antioxidant enzyme TXN and decreases the sFlt release [150]. In placental explant models with melatonin treatment, lower oxidative stress (8-isoprene) and higher antioxidant markers (Nrf2 transposition, HO-1) were observed, but the secretion of anti-angiogenic factors (sFlt, sEng and activin A) remained steady. Similarly, melatonin reduces the expression of vascular cell adhesion molecules induced by TNF-α and upregulates both antioxidant TXN and GCLC expression in human umbilical vein endothelial cells (HUVECs) without altering the secretion of sFlt or sEng. Melatonin has shown its safety and efficacy for both mothers and fetuses in a phase I clinical trial, leading to extended delivery interval by 6 ± 2.3 days, and the less requirement of antihypertensive medication on days 3–4 decreased from 71 to 13%, days 6–7 dropped from 51 to 8%, and at delivery reduced from 75 to 26% [151]. Hence, melatonin can be a possible adjuvant therapy to prolong the pregnancy time and improve the clinical results of PE [151].

**Fig. 4:**
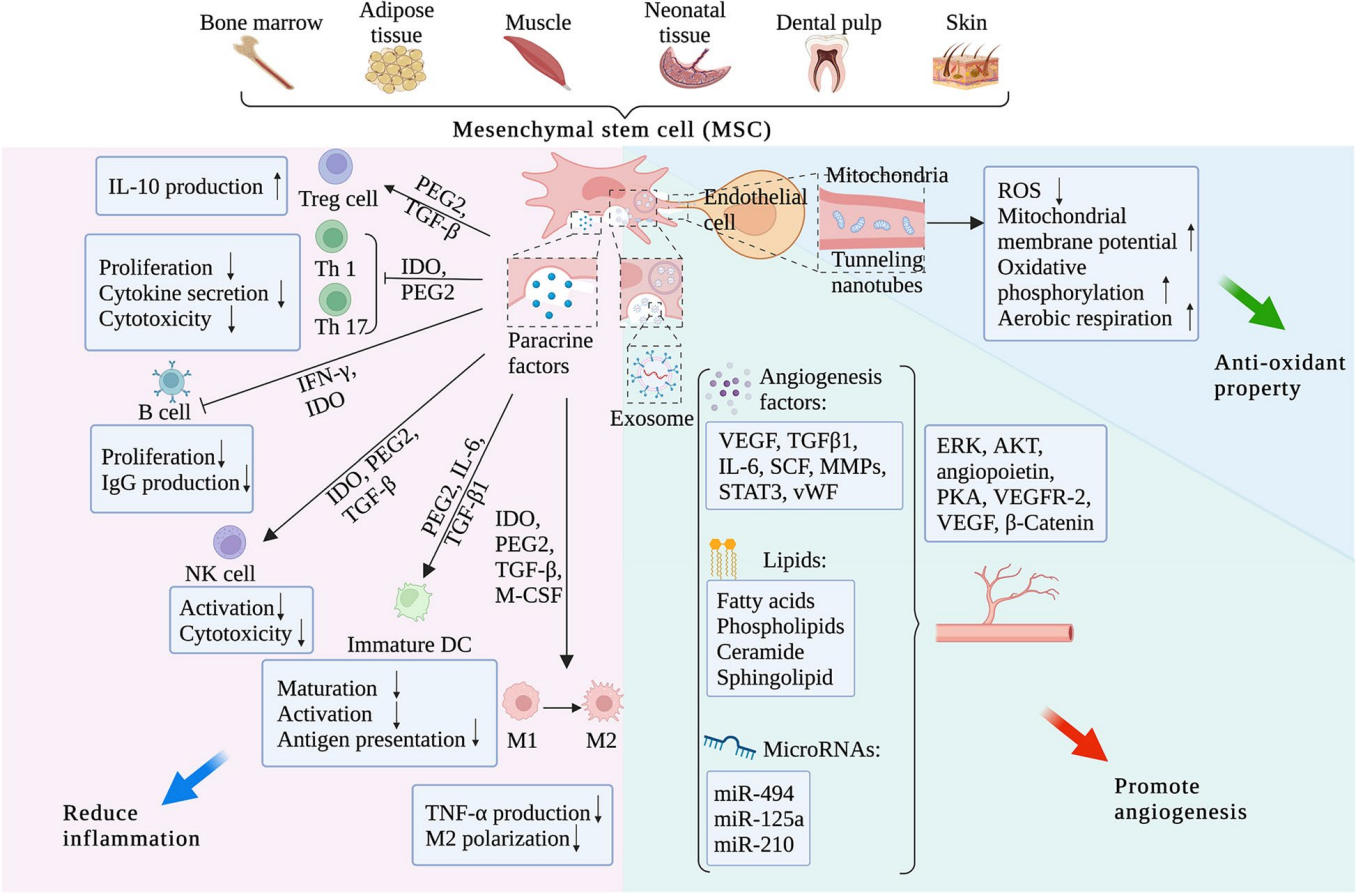
Mesenchymal stem cell (MSC) therapeutic effect on preeclampsia (PE). The therapeutic effect of mesenchymal stem cells (MSCs) on PE has been investigated, mainly including anti-inflammation, pro-angiogenesis and anti-oxidance. MSCs possess abundant sources such as bone marrow, adipose tissue, muscle, neonatal tissue and skin. First, MSCs can secrete a series of paracrine factors, including PEG2, TGF-β, IFN-γ and M-CSF, target various immune cells such as T lymphocytes, B lymphocytes, dendritic cells and natural killer cells. Then, the cytokine profile, maturation, polarization and activation of target cells will be regulated, suppressing the inflammation occurring in PE. Second, the MSC-derived exosomes have raised great attention due to its ability to transport angiogenesis factors, lipids and microRNAs. As the regulatory factors transfering biologically active membrane and cytosolic components to target cells, the dysfunctional intercellular communication can be improved. In PE, the application of MSC-derived exosomes alleviates the degenerative angiogenesis through regulating the levels of ERK, AKT, angiopoietin, PKA, VEGFR-2, VEGF and β-catenin. Third, MSCs possess the anti-oxidant capability though transferring functional mitochondria to the target cells in affected tissues of PE patients. As a result, the mitochondrial ROS can be reduced, mitochondrial membrane potential and oxidative phosphorylation levels in recipient cells will be restored. Also, aerobic respiration will be rescued and the apopotosis of endothelial cells will be inhibited to alleviate oxitant stress raised by PE. (Abbreviation: AKT, RAC(Rho family)-alpha serine/threonine-protein kinase; ERK, extracellular-signal regulated kinase; IDO, indoleamine 2,3-dioxygenase; IFN-γ, interferon gamma; IgG, immunoglobulin G; L-10, interleukin 10; M-CSF, macrophage colony-stimulating factor; miR, microRNA; PEG2, prostaglandin E2; PKA, cyclic-AMP cascade protein kinase A; ROS, reactive oxygen species; Th, T helper cell; TGF-β, transforming growth factor beta; VEGF, vascular endothelial growth factor; VEGFR-2, vascular endothelial growth factor receptor-2)

**Mesenchymal stem cells (MSCs)** have received increasing attention for PE treatment due to their low immunogenicity, powerful immunomodulation, angiogenesis and regenerative therapy, as shown in Fig. 4 [152]. Bone marrow is the main source of MSCs, but the extraction process is invasive, and the number of MSCs decreases with age, which limits the clinical application of MSCs [153]. Human cord blood has been found to be rich in MSCs, whose characteristics are similar to those of bone marrow MSCs [154]. Therefore, the MSCs derived from placenta or umbilical cord may have the potential to treat endotoxin-induced hypertension in patients with PE. Studies have shown that placental derived MSCs have great therapeutic potential in PE ischemia [155, 156]. In vitro culture, MSCs have successfully differentiated into polysyncytic trophoblasts and HLA-G + extra-villous trophoblasts [157]. During PE development, inflammatory Th1 cells would exacerbate immune imbalances and reduce the number of regulatory T cells and anti-inflammatory cytokines, leading to chronic inflammation and associated oxidative stress. In this case, MSCs can significantly improve the symptoms of Th1 cells of PE mouse model, implying that MSCs may play a significant role in maternal interface immunity [158]. Consistent with these, another study demonstrated that MSCs could improve the functions of mouse trophoblasts and endothelial cells to promote angiogenesis through attenuating the hypoxia-induced mitochondria damage [159]. As for the underlying mechanisms, there is increasing evidence that the beneficial effects of MSCs are exerted through the secretory bodies and reducing SFLT-1 to restore angiogenesis. Additionally, human placental mesenchymal-like adherent stromal cells have been found to secrete IL-6 and VEGF to confer neuroprotection to nerve growth factor-differentiated PC12 cells exposed to ischemia [160–162]. Recently, Todd et al. demonstrated the mechanisms of FKBPL in MSC-mediated angiogenic and anti-inflammatory effects [163]. MSCs enhanced trophoblast migration and endothelial tubule formation under hypoxia and normoxia, which associated with decreased levels of anti-angiogenic protein FKBPL and increased expression of pro-angiogenic CD44 mRNA. Besides inhibiting angiogenesis, FKBPL is involved in the regulation of inflammatory pathways, such as STAT3 [164], which are associated with trophoblast functions [165, 166]. Thus, MSCs may improve the vascular response and symptoms of PE by restoring normal levels of FKBPL. Despite such promising results, a series of questions of MSC therapy in PE, including cellular quality and dosage control, need to be addressed prior to a clinical setting.

The MSC-derived exosomes have raised great attention due to its ability to transport angiogenesis factors, lipids and microRNAs. As the regulatory factors transfer biologically active membrane and cytosolic components to target cells, the dysfunctional intercellular communication can be improved. In PE, the application of MSC-derived exosomes alleviatesdegenerative angiogenesis through regulating the levels of ERK, AKT, angiopoietin, PKA, VEGFR-2, VEGF and β-catenin. MicroRNAs (miRNAs) are small (21–23 nucleotides) single-stranded RNA molecules that play an important role in the post-transcriptional regulation of gene expression by promoting RNA instability or translation inhibition [167, 168]. The miRNAs in exosomes have been considered to characterize the disease progression, and thus can be used as novel biomarkers to predict PE [169, 170]. In addition, exosomes of human umbilical cord mesenchymal stem cells (uc-MSCs) have been reported to protect ovarian granulosa cells from cisplatin-induced damage and chemotherapy-induced apoptosis in vitro [171]. MiR-133b, a member of big miRNA family, is downregulated in PE though targeting serum and glucocorticoid-regulated kinase 1 (SGK1) [172, 173]. SGK1 is a serine/threonine kinase that plays a restrictive role in regulating cell survival, proliferation and differentiation. Kong et al. applied the trophoblast cell HPT-8 and HTR8-S/Vneo co-cultured with human uc-MSC-derived exosomes that had been transfected with miR-133b plasmids [174]. Accordingly, miR-133b is down-regulated and SGK1 is up-regulated in PE groups, and miR-133b derived from exosomes in uc-MSCs facilitates trophoblast cell proliferation, migration and invasion in PE via constraining SGK1. Two recent studies have shown that the up-regulation of placental miR-210 is a hallmark of severe PE [175, 176]. MiR-210 is a primary miRNA of hypoxic response and a common component of tumor microenvironment, that can be induced in a hypoxia-inducible factor (HIF)-1α dependent manner [177]. Immunoprecipitation analysis showed that HIF-1α regulates miR-210 expression in a variety of tumors. Lee et al. suggested that the iron-sulfur cluster scaffold homologue down-regulated by miR-210 perturbing trophoblast iron metabolism is associated with defective placentation of PE [177], indicating that miR-210 is promising to treat PE.

Currently, the oncogenicity of MSC therapy has turned researchers’ attention to the MSC-derived exosome treatment. The exosome, a subtype of extracellular vesicle, possesses lower susceptibility to damage by hostile injury microenvironment (e.g. high concentrations of cytokines and hypoxia) than that of MSCs. Additionally, the exosomes can retain their efficacy after freezing, and thus the costly stem cell facilities are no need for MSC-exosome production. Nevertheless, in contrast to MSCs, the nonrenewable exosome may be used up in vivo. In clinical applications, the repeated injection line and procedure of MSC-exosome in specific phases may be considered.

## Discussion

PE is a syndrome with various phenotypes, with long-term effects on the fetus and mother including the an increased possibility of adult disease in fetus and a larger risk of cardiovascular disease in mother after pregnancy. There is an urgent need to identify PE in early stage in order to achieve their specific treatment. Such studies require large-scale samples, which can be achieved by setting rules of standardized and coordinated collection of clinical data and biological specimens. Future research should include samples scaled globally, and cutting-edge methods such as machine learning and artificial intelligence can be considered to collect and analyze data. The objective is to determine the precise early PE prediction and the implementation of phenotypic specific therapies.

Currently, the only effective treatment for PE is childbirth, but new treatments are being developed to reduce complications and prolong pregnancy. Aspirin has been recommended as a preventive therapy for PE in preterm labor, and statins are being explored as another potential intervention. In the future, it may be possible to target PE therapy by using pro-angiogenic factors or removing anti-angiogenic factors to restore angiogenic balance. MSCs provide a promising tool for future therapeutic applications because of their anti-inflammatory, pro-angiogenic, antioxidant and immunomodulatory potentials, as well as their easy isolation, great self-renewal ability and lack of immunogenicity. MSCs can be obtained from different tissue sources and vary in differentiation stage, proteome, and genome profile, achieving different functions. Although several molecular pathways have been identified, the intact mechanisms by which MSCs play a therapeutic role in PE are still not fully understood [152]. According to Zhang et al., direct cell-to-cell contact was insignificant during MSC treatment [178]. Given the pathogenesis of PE, each secreted molecule from MSCs plays a partial role in restoring normal functions. In the future, the exact molecules can be identified by inhibiting or knocking them out individually or simultaneously, and their therapeutic potential need to be investigated. There are some issues that need to be addressed in bringing MSC therapy from in vivo PE studies into clinical trials. Currently, no clinical trial of long-term exposure to MSCs has been reported and there is a lack of complete examination of the effects of MSCs on fetal and maternal health [179]. Due to their regenerative and angiogenic effects, MSCs may promote malignancy which needs to be fully studied prior to treatment [152]. In general, standardized procedures of isolation and propagation need to be developed, which will maximize the therapeutic potential and minimize the side effects of MSC therapy.

While various drugs show great potential in preclinical practice, the off-target effects remain of concern when deliver to a pregnant woman, as they can harm a developing fetus [180]. Nanoparticles, a targeted delivery system, can prevent the off-target effects by delivering therapeutic drugs to specific cells, tissues or organs. Nanoparticles are similar in size to proteins, usually less than 100 nm [181], and can facilitate their functions by leveraging existing cellular mechanisms [182]. Nanoparticles can be targeted to epidermal growth factor receptor, which is expressed about 3000 times more in placenta than in other tissues [183]. Nanoparticles can be designed to cross the placenta easily into the fetal chamber, or modified to deliberately prevent them from crossing the placenta, or synthesized so that the placenta is the site of action [184]. Nanoparticles tend to have longer half-lives and thus, the effective doses are commonly lower than therapies used alone.

An array of types of nanoparticles are promising for PE treatment, including liposomes, polymers, dendritic molecules and inorganic nanoparticles. Liposomes are vesicular bimolecular layers synthesized from phospholipids, which can increase the solubility of drugs [185], but their low loading capacity may lead to rapid drug release and unstable storage [182]. Polymer nanoparticles can be synthesized from natural or biodegradable synthetic materials and can change their physical or chemical properties in response to environmental signals [182]. Dendrite molecules are larger synthetic nanoparticles, usually with a dendritic structure, strong loading capacity, drug sitting on the external branch, and predictable pharmacokinetics [186]. Inorganic nanoparticles, such as gold and iron oxide, have a large load capacity and low toxicity [187], but they have not yet been explored for use in pregnancy due to their unclear safety profile. Emerging treatments have used target delivery therapy. For instance, bacteria-derived organic nanoparticles have been used to deliver doxorubicin into placental tissue as a non-invasive treatment for ectopic pregnancy [188]. Magnetic iron oxide nanoparticles have been used in a similar approach using magnetic targeting instead of EGFR targeting [189]. Generally, combining drugs used to treat PE with novel nanoparticle targeted delivery systems may further improve drug efficacy and reduce off-target effects.

One of the challenges of PE research is their reproducibility in animal models, because spontaneous PE is exclusive to human pregnancy, making it difficult to replicate in animal models accurately. At present, cutting the abdominal aorta and uterine aorta in pregnant rats is one of the most common models, appearing the symptoms including systolic hypertension, increased renal endothelial cells and proteinuria [190]. Besides, a transgenic blastocyst with a trophoblast ectoderm expressing human sFlt1 via lentiviral vectors has been implanted [191]. During the pregnancy progress, human sFlt1 level increases in maternal serum, with the corresponding development of PE features. However, these rodents failed to develop all severe PE features as humans do. In addition, genetic rodent models have been developed. For instance, the BPH/5 model showed elevated MAP, proteinuria, progressive glomerular damage, and a significant decrease in birth rate during the third trimester of pregnancy [192]. COMT knockout mouse models showed similar systolic hypertension, increased sFlt1 level, and decreased proteinuria after delivery, but there was no evidence of systemic vascular injury or fetal growth restriction, suggesting that the model closed to gestational hypertension or mild PE [193]. Based on the observation that human placenta chemically induces systemic hypertension, an old rodent model has been applied to simulate gestational hypertension by manipulating the renin-angiotensin system [194]. Although in human, PE is characterized by inhibition of renin and angiotensin II, this animal model can mimic the molecular environment of uteroplacental hypoperfusion and various cytokines and proteins released into the maternal circulation. Following these, animal models can extend the relevant data observed in human to causal relationships and can be a key tool for assessing toxicity and efficacy of new therapies. Of course, animal models for this human-specific disease are imperfect, as they cannot prove thrombocytopenia, HELLP syndrome, eclampsia and other signs and symptoms that define the severe features of PE in human [23]. The main drivers of PE, names inadequate trophoblast infiltration and failure of spiral artery remodeling, have not been accurately modeled [195]. Since the gestation period of humans is significantly longer than that of rodents, animal models need to be exposed to higher doses of circulating toxins. Interestingly, studies have found that rodents did develop severe PE characteristics when the levels of sFlt1 and sEng were increased [196–198]. With the development of tissue engineering, 3-dimentional in vitro models have attracted various researches due to the lack of reliable animal models. To ensure a good resemble of complex in vivo microenvironment, scaffold materials, seeding cells, and growth factors are significant for cell–cell interactions and cell–matrix interactions [199]. Haider et al. constructed a long-term expanding organoid from purified first-trimester cytotrophoblasts embedded in Matrigel containing a mixture of growth factors and signaling inhibitors including epidermal growth factor, Noggin and R-spondin [200]. To further research the underlying mechanisms and appropriate managements of PE, innovative experimental models are needed.

## Conclusion

The emergence of multiple biomarkers with accurate test outcomes provides multi-dimensional targeting to complete the antenatal and postnatal care of PE, aiming towards ultra-rapid bedside prediction of women with the suspicion of PE. Various biochemical factors combined with improved therapeutic approaches, which have been displayed to be valuable and expandable into widespread clinical practice, will be integrated into the management standard of patients with suspected or confirmed PE over the coming years.

## Data Availability

The data and material that support this review are openly available.

## References

[1] Say L, Chou D, Gemmill A, Tunçalp Ö, Moller AB, Daniels J, Gülmezoglu AM, Temmerman M, Alkema L (2014). Global causes of maternal death: a WHO systematic analysis. Lancet Glob Health.

[2] Brown HL (2017). Medical and Surgical Innovations in Health Care. Obstet Gynecol.

[3] Redman CW, Sargent IL, Staff AC (2014). IFPA Senior Award Lecture: making sense of pre-eclampsia - two placental causes of preeclampsia?. Placenta.

[4] Redman CW, Sargent IL (2005). Latest advances in understanding preeclampsia. Science.

[5] Romero R, Chaiworapongsa T (2013). Preeclampsia: a link between trophoblast dysregulation and an antiangiogenic state. J Clin Invest.

[6] Palei AC, Spradley FT, Warrington JP, George EM, Granger JP (2013). Pathophysiology of hypertension in pre-eclampsia: a lesson in integrative physiology. Acta Physiol (Oxf).

[7] Huppertz B (2008). Placental origins of preeclampsia: challenging the current hypothesis. Hypertension.

[8] Ives CW, Sinkey R, Rajapreyar I, Tita ATN, Oparil S (2020). Preeclampsia-Pathophysiology and Clinical Presentations: JACC State-of-the-Art Review. J Am Coll Cardiol.

[9] Goel A, Maski MR, Bajracharya S, Wenger JB, Zhang D, Salahuddin S, Shahul SS, Thadhani R, Seely EW, Karumanchi SA, Rana S (2015). Epidemiology and Mechanisms of De Novo and Persistent Hypertension in the Postpartum Period. Circulation.

[10] Ramos JGL, Sass N, Costa SHM (2017). Preeclampsia. Rev Bras Ginecol Obstet.

[11] Poon LC, Kametas NA, Chelemen T, Leal A, Nicolaides KH (2010). Maternal risk factors for hypertensive disorders in pregnancy: a multivariate approach. J Hum Hypertens.

[12] Wright D, Syngelaki A, Akolekar R, Poon LC, Nicolaides KH (2015). Competing risks model in screening for preeclampsia by maternal characteristics and medical history. Am J Obstet Gynecol.

[13] Poon LC, Kametas NA, Pandeva I, Valencia C, Nicolaides KH (2008). Mean arterial pressure at 11(+0) to 13(+6) weeks in the prediction of preeclampsia. Hypertension.

[14] Jasović-Siveska E, Jasović V (2011). Prediction of mild and severe preeclampsia with blood pressure measurements in first and second trimester of pregnancy. Ginekol Pol.

[15] A Ridder, V Giorgione, A Khalil, B Thilaganathan. Preeclampsia: The Relationship between Uterine Artery Blood Flow and Trophoblast Function. Int J Mol Sci. 2019;20(13). 10.3390/ijms20133263 PMC6651116 31269775

[16] Plasencia W, Maiz N, Bonino S, Kaihura C, Nicolaides KH (2007). Uterine artery Doppler at 11 + 0 to 13 + 6 weeks in the prediction of pre-eclampsia. Ultrasound Obstet Gynecol.

[17] Plasencia W, Maiz N, Poon L, Yu C, Nicolaides KH (2008). Uterine artery Doppler at 11 + 0 to 13 + 6 weeks and 21 + 0 to 24 + 6 weeks in the prediction of pre-eclampsia. Ultrasound Obstet Gynecol.

[18] Poon LC, Staboulidou I, Maiz N, Plasencia W, Nicolaides KH (2009). Hypertensive disorders in pregnancy: screening by uterine artery Doppler at 11–13 weeks. Ultrasound Obstet Gynecol.

[19] Sachan R, Patel ML, Sachan P, Shyam R, Vandana (2021). Role of platelet count and mean platelet volume and red cell distribution width in the prediction of preeclampsia in early pregnancy. J Family Med Prim Care..

[20] Viana-Rojas JA, Rosas-Cabral A, Prieto-Macías J, Terrones-Saldívar MC, Arcos-Noguez P, Bermúdez-Gómez J, Martínez-Padilla LE, Sandoval-Valdez DA, Hernández-González F, Serrano-Díaz LC (2017). Relation of red cell distribution width and mean platelet volume with the severity of preeclampsia. Rev Med Inst Mex Seguro Soc.

[21] Kim H, Lee JU, Kim S, Song S, Sim SJ (2019). A Nanoplasmonic Biosensor for Ultrasensitive Detection of Alzheimer's Disease Biomarker Using a Chaotropic Agent. ACS Sens.

[22] Williams MD, Wheby MS (1992). Anemia in pregnancy. Med Clin North Am.

[23] Rana S, Lemoine E, Granger JP, Karumanchi SA (2019). Preeclampsia: Pathophysiology, Challenges, and Perspectives. Circ Res.

[24] Hypertension in pregnancy (2013). Report of the American College of Obstetricians and Gynecologists’ Task Force on Hypertension in Pregnancy. Obstet Gynecol.

[25] Quitterer U, Fu X, Pohl A, Bayoumy KM, Langer A, AbdAlla S (2019). Beta-Arrestin1 Prevents Preeclampsia by Downregulation of Mechanosensitive AT1-B2 Receptor Heteromers. Cell.

[26] Sahraravand M, Järvelä IY, Laitinen P, Tekay AH, Ryynänen M (2011). The secretion of PAPP-A, ADAM12, and PP13 correlates with the size of the placenta for the first month of pregnancy. Placenta.

[27] Huppertz B, Meiri H, Gizurarson S, Osol G, Sammar M (2013). Placental protein 13 (PP13): a new biological target shifting individualized risk assessment to personalized drug design combating pre-eclampsia. Hum Reprod Update.

[28] Nicolaides KH, Bindra R, Turan OM, Chefetz I, Sammar M, Meiri H, Tal J, Cuckle HS (2006). A novel approach to first-trimester screening for early pre-eclampsia combining serum PP-13 and Doppler ultrasound. Ultrasound Obstet Gynecol.

[29] N Moslemi Zadeh, F Naghshvar, S Peyvandi, P Gheshlaghi, S Ehetshami. PP13 and PAPP-A in the First and Second Trimesters: Predictive Factors for Preeclampsia?, ISRN Obstet Gynecol. 2012 (2012):263871. 10.5402/2012/263871 PMC3385696 22778981

[30] Goetzinger KR, Zhong Y, Cahill AG, Odibo L, Macones GA, Odibo AO (2013). Efficiency of first-trimester uterine artery Doppler, a-disintegrin and metalloprotease 12, pregnancy-associated plasma protein a, and maternal characteristics in the prediction of preeclampsia. J Ultrasound Med.

[31] Park HJ, Shim SS, Cha DH (2015). Combined Screening for Early Detection of Pre-Eclampsia. Int J Mol Sci.

[32] Zhang X, Huangfu Z, Shi F, Xiao Z (2021). Predictive Performance of Serum β-hCG MoM Levels for Preeclampsia Screening: A Meta-Analysis. Front Endocrinol (Lausanne).

[33] Hasija A, Balyan K, Debnath E, Kumar RVM (2021). Prediction of hypertension in pregnancy in high risk women using maternal factors and serial placental profile in second and third trimester. Placenta.

[34] Hodická Z, Bienertová-Vasků J, Ventruba P, Vasků A (2012). Adrenocorticotropin hormone–possible marker of pregnancy pathologies. Ceska Gynekol.

[35] Lamarca B (2012). Endothelial dysfunction An important mediator in the pathophysiology of hypertension during pre-eclampsia, Minerva. Ginecol.

[36] Mistry HD, Ogalde MVH, Broughton Pipkin F, Escher G, Kurlak LO (2020). Maternal, Fetal, and Placental Selectins in Women With Pre-eclampsia; Association With the Renin-Angiotensin-System. Front Med (Lausanne)..

[37] Akhter T, Wikström AK, Larsson M, Larsson A, Wikström G, Naessen T (2017). Serum Pentraxin 3 is associated with signs of arterial alteration in women with preeclampsia. Int J Cardiol.

[38] Colmenares-Mejía CC, Quintero-Lesmes DC, Bautista-Niño PK, Guio Mahecha E, Beltrán Avendaño M, Díaz Martínez LA, Ortiz Serrano R, Páez Leal MC, Monterrosa Castro Á, Mesa Restrepo CM, Monsalve G, Sanín-Blair E, Saldarriaga W, Luna ML, Casas JP, Serrano Díaz N (2020). Pentraxin-3 is a candidate biomarker on the spectrum of severity from pre-eclampsia to HELLP syndrome: GenPE study. Hyperten Res..

[39] Németh B, Murányi E, Hegyi P, Mátrai P, Szakács Z, Varjú P, Hamvas S, Tinusz B, Budán F, Czimmer J, Bérczi B, Erőss B, Gyöngyi Z, Kiss I (2018). Asymmetric dimethylarginine levels in preeclampsia - Systematic review and meta-analysis. Placenta.

[40] Akhter T, Wikström G, Larsson M, Bondesson U, Hedeland M, Naessen T (2021). Dimethylarginines correlate to common carotid artery wall layer dimensions and cardiovascular risk factors in pregnant women with/without preeclampsia: A group comparative study. Eur J Obstet Gynecol Reprod Biol.

[41] Garg P, Jaryal AK, Kachhawa G, Deepak KK, Kriplani A (2018). Estimation of asymmetric dimethylarginine (ADMA), placental growth factor (PLGF) and pentraxin 3 (PTX 3) in women with preeclampsia. Pregnancy Hypertens.

[42] Gomes H, Cabral ACV, Andrade SP, Leite HV, Teixeira PG, Campos PP, Gomes JAA (2020). Cystatin C as an indicator of renal damage in pre-eclampsia. Hypertens Pregnancy.

[43] Stout MJ, Conner SN, Colditz GA, Macones GA, Tuuli MG (2015). The Utility of 12-Hour Urine Collection for the Diagnosis of Preeclampsia: A Systematic Review and Meta-analysis. Obstet Gynecol.

[44] Lindheimer MD, Kanter D (2010). Interpreting abnormal proteinuria in pregnancy: the need for a more pathophysiological approach. Obstet Gynecol.

[45] Valdés E, Sepúlveda-Martínez Á, Tong A, Castro M, Castro D (2016). Assessment of Protein: Creatinine Ratio versus 24-Hour Urine Protein in the Diagnosis of Preeclampsia. Gynecol Obstet Invest.

[46] Zou L-X, Sun L, Nicholas SB, Lu Y, K SS, Hua R (2020). Comparison of bias and accuracy using cystatin C and creatinine in CKD-EPI equations for GFR estimation. Eur J Intern Med..

[47] Risch M, Purde MT, Baumann M, Mohaupt M, Mosimann B, Renz H, Raio L, Surbek D, Risch L (2017). High first-trimester maternal blood cystatin C levels despite normal serum creatinine predict pre-eclampsia in singleton pregnancies. Scand J Clin Lab Invest.

[48] HB Zhang, JM Fan, LL Zhu, XH Yuan, XW Shen. Combination of NGAL and Cystatin C for Prediction of Preeclampsia at 10–14 Weeks of Gestation. Clin Lab. 2019;65(5) 10.7754/Clin.Lab.2018.180831 31115219

[49] Park YS, Kim Y, Kim HY, Ahn KH, Cho GJ, Hong SC, Oh MJ, Kim HJ (2020). Serum sFlt-1, cystatin C and cathepsin B are potential severity markers in preeclampsia: a pilot study. Arch Gynecol Obstet.

[50] Chen DB, Zheng J (2014). Regulation of placental angiogenesis. Microcirculation.

[51] Karumanchi SA (2016). Angiogenic Factors in Preeclampsia: From Diagnosis to Therapy. Hypertension.

[52] Eddy AC, Bidwell GL, George EM (2018). Pro-angiogenic therapeutics for preeclampsia. Biol Sex Differ.

[53] Rădulescu C, Bacârea A, Huțanu A, Gabor R, Dobreanu M (2016). Placental Growth Factor, Soluble fms-Like Tyrosine Kinase 1, Soluble Endoglin, IL-6, and IL-16 as Biomarkers in Preeclampsia. Mediators Inflamm.

[54] Zeisler H, Llurba E, Chantraine F, Vatish M, Staff AC, Sennström M, Olovsson M, Brennecke SP, Stepan H, Allegranza D, Dilba P, Schoedl M, Hund M, Verlohren S (2016). Predictive Value of the sFlt-1:PlGF Ratio in Women with Suspected Preeclampsia. N Engl J Med.

[55] Nabweyambo S, Sande OJ, McGovern N, Bwanga F, Ssekagiri A, Keesiga A, Adroma M, Wasswa R, Atuheirwe M, Namugenyi J, Castelnuovo B, Nakimuli A (2021). Circulating levels of angiogenic factors and their association with preeclampsia among pregnant women at Mulago National Referral Hospital in Uganda. PLoS ONE.

[56] W Duan, C Xia, K Wang, Y Duan, P Cheng, B Xiong. A meta-analysis of the vascular endothelial growth factor polymorphisms associated with the risk of pre-eclampsia. Biosci Rep. 2020;40(5) 10.1042/BSR20190209 PMC7240197 32255175

[57] Pacheco Romero J, Acosta O, Huerta D, Cabrera S, Vargas M, Mascaro P, Huamán M, Sandoval J, López R, Mateus J, Gil E, Guevara E, Butrica N, Catari D, Bellido D, Custodio G, Naranjo A (2021). Genetic markers for preeclampsia in Peruvian women. Colomb Med..

[58] Xu YT, Shen MH, Jin AY, Li H, Zhu R (2017). Maternal circulating levels of transforming growth factor-β superfamily and its soluble receptors in hypertensive disorders of pregnancy. Int J Gynaecol Obstet.

[59] Giguère Y, Charland M, Bujold E, Bernard N, Grenier S, Rousseau F, Lafond J, Légaré F, Forest JC (2010). Combining biochemical and ultrasonographic markers in predicting preeclampsia: a systematic review. Clin Chem.

[60] Hahn S, Rusterholz C, Hösli I, Lapaire O (2011). Cell-free nucleic acids as potential markers for preeclampsia. Placenta.

[61] Chan KC, Ding C, Gerovassili A, Yeung SW, Chiu RW, Leung TN, Lau TK, Chim SS, Chung GT, Nicolaides KH, Lo YM (2006). Hypermethylated RASSF1A in maternal plasma: A universal fetal DNA marker that improves the reliability of noninvasive prenatal diagnosis. Clin Chem.

[62] Kim SY, Kim HJ, Park SY, Han YJ, Choi JS, Ryu HM (2016). Early Prediction of Hypertensive Disorders of Pregnancy Using Cell-Free Fetal DNA Cell-Free Total DNA, and Biochemical Markers. Fetal Diagn Ther.

[63] Saraswathy S, Sahai K, Yadav TP, Arora D, Mendiratta SL, Naqvi SH, Biswas S, Krishnan M, Abraham KM (2016). Evaluation of fetal hypermethylated RASSF1A in pre-eclampsia and its relationship with placental protein-13, pregnancy associated plasma protein-A and urine protein. Pregnancy Hypertens.

[64] Salvianti F, Inversetti A, Smid M, Valsecchi L, Candiani M, Pazzagli M, Cremonesi L, Ferrari M, Pinzani P, Galbiati S (2015). Prospective evaluation of RASSF1A cell-free DNA as a biomarker of pre-eclampsia. Placenta.

[65] Karapetian AO, Baev OR, Sadekova AA, Krasnyi AM, Sukhikh GT (2021). Cell-Free Foetal DNA as a Useful Marker for Preeclampsia Prediction. Reprod Sci.

[66] Smid M, Galbiati S, Lojacono A, Valsecchi L, Platto C, Cavoretto P, Calza S, Ferrari A, Ferrari M, Cremonesi L (2006). Correlation of fetal DNA levels in maternal plasma with Doppler status in pathological pregnancies. Prenat Diagn.

[67] Kim HJ, Kim SY, Lim JH, Kwak DW, Park SY, Ryu HM (2015). Quantification and Application of Potential Epigenetic Markers in Maternal Plasma of Pregnancies with Hypertensive Disorders. Int J Mol Sci.

[68] Hromadnikova I, Zejskova L, Kotlabova K, Jancuskova T, Doucha J, Dlouha K, Krofta L, Jirasek JE, Vlk R (2010). Quantification of extracellular DNA using hypermethylated RASSF1A, SRY, and GLO sequences–evaluation of diagnostic possibilities for predicting placental insufficiency. DNA Cell Biol.

[69] Martin A, Krishna I, Martina B, Samuel A (2014). Can the quantity of cell-free fetal DNA predict preeclampsia: a systematic review. Prenat Diagn.

[70] Phipps EA, Thadhani R, Benzing T, Karumanchi SA (2019). Pre-eclampsia: pathogenesis, novel diagnostics and therapies. Nat Rev Nephrol.

[71] Pennington KA, Schlitt JM, Jackson DL, Schulz LC, Schust DJ (2012). Preeclampsia: multiple approaches for a multifactorial disease. Dis Model Mech.

[72] Mol BWJ, Roberts CT, Thangaratinam S, Magee LA, de Groot CJM, Hofmeyr GJ (2016). Pre-eclampsia. Lancet.

[73] Burton GJ, Redman CW, Roberts JM, Moffett A (2019). Pre-eclampsia: pathophysiology and clinical implications. BMJ.

[74] Moufarrej MN, Vorperian SK, Wong RJ, Campos AA, Quaintance CC, Sit RV, Tan M, Detweiler AM, Mekonen H, Neff NF, Baruch-Gravett C, Litch JA, Druzin ML, Winn VD, Shaw GM, Stevenson DK, Quake SR (2022). Early prediction of preeclampsia in pregnancy with cell-free RNA. Nature.

[75] Rasmussen M, Reddy M, Nolan R, Camunas-Soler J, Khodursky A, Scheller NM, Cantonwine DE, Engelbrechtsen L, Mi JD, Dutta A, Brundage T, Siddiqui F, Thao M, Gee EPS, La J, Baruch-Gravett C, Santillan MK, Deb S, Ame SM, Ali SM, Adkins M, DePristo MA, Lee M, Namsaraev E, Gybel-Brask DJ, Skibsted L, Litch JA, Santillan DA, Sazawal S, Tribe RM, Roberts JM, Jain M, Høgdall E, Holzman C, Quake SR, Elovitz MA, McElrath TF (2022). RNA profiles reveal signatures of future health and disease in pregnancy. Nature.

[76] Han X, Ghaemi MS, Ando K, Peterson LS, Ganio EA, Tsai AS, Gaudilliere DK, Stelzer IA, Einhaus J, Bertrand B, Stanley N, Culos A, Tanada A, Hedou J, Tsai ES, Fallahzadeh R, Wong RJ, Judy AE, Winn VD, Druzin ML, Blumenfeld YJ, Hlatky MA, Quaintance CC, Gibbs RS, Carvalho B, Shaw GM, Stevenson DK, Angst MS, Aghaeepour N, Gaudilliere B (2019). Differential Dynamics of the Maternal Immune System in Healthy Pregnancy and Preeclampsia. Front Immunol.

[77] SE Ander, MS Diamond, CB Coyne. Immune responses at the maternal-fetal interface. Sci Immunol. 2019;4(31) 10.1126/sciimmunol.aat6114 PMC6744611 30635356

[78] Ibarra A, Zhuang J, Zhao Y, Salathia NS, Huang V, Acosta AD, Aballi J, Toden S, Karns AP, Purnajo I, Parks JR, Guo L, Mason J, Sigal D, Nova TS, Quake SR, Nerenberg M (2020). Non-invasive characterization of human bone marrow stimulation and reconstitution by cell-free messenger RNA sequencing. Nat Commun.

[79] Pillay P, Moodley K, Moodley J, Mackraj I (2017). Placenta-derived exosomes: potential biomarkers of preeclampsia. Int J Nanomedicine.

[80] K Matsubara, Y Matsubara, Y Uchikura, T Sugiyama. Pathophysiology of Preeclampsia: The Role of Exosomes. Int J Mol Sci. 2021;22(5) 10.3390/ijms22052572 PMC7961527 33806480

[81] Gao X, Shao L, Ge X, Zhang L, Chen D, He R (2020). The Potential Role of Serum Exosomes in Preeclampsia. Curr Drug Metab.

[82] Chang X, Yao J, He Q, Liu M, Duan T, Wang K (2018). Exosomes From Women With Preeclampsia Induced Vascular Dysfunction by Delivering sFlt (Soluble Fms-Like Tyrosine Kinase)-1 and sEng (Soluble Endoglin) to Endothelial Cells. Hypertension.

[83] Tan KH, Tan SS, Ng MJ, Tey WS, Sim WK, Allen JC, Lim SK (2017). Extracellular vesicles yield predictive pre-eclampsia biomarkers. J Extracell Vesicles.

[84] Pillay P, Moodley K, Vatish M, Moodley J, Duarte R, Mackraj I (2020). Exosomal Th1/Th2 cytokines in preeclampsia and HIV-positive preeclamptic women on highly active anti-retroviral therapy. Cytokine.

[85] Yao X, Wei W, Wang X, Chenglin L, Björklund M, Ouyang H (2019). Stem cell derived exosomes: microRNA therapy for age-related musculoskeletal disorders. Biomaterials.

[86] Li H, Ouyang Y, Sadovsky E, Parks WT, Chu T, Sadovsky Y (2020). Unique microRNA Signals in Plasma Exosomes from Pregnancies Complicated by Preeclampsia. Hypertension.

[87] Devor E, Santillan D, Scroggins S, Warrier A, Santillan M (2020). Trimester-specific plasma exosome microRNA expression profiles in preeclampsia. J Matern Fetal Neonatal Med.

[88] Han L, Luo QQ, Peng MG, Zhang Y, Zhu XH (2021). miR-483 is downregulated in pre-eclampsia via targeting insulin-like growth factor 1 (IGF1) and regulates the PI3K/Akt/mTOR pathway of endothelial progenitor cells. J Obstet Gynaecol Res.

[89] Tang Q, Gui J, Wu X, Wu W (2019). Downregulation of miR-424 in placenta is associated with severe preeclampsia. Pregnancy Hypertens.

[90] Cui J, Chen X, Lin S, Li L, Fan J, Hou H, Li P (2020). MiR-101-containing extracellular vesicles bind to BRD4 and enhance proliferation and migration of trophoblasts in preeclampsia. Stem Cell Res Ther.

[91] Wang Z, Wang P, Wang Z, Qin Z, Xiu X, Xu D, Zhang X, Wang Y (2019). MiRNA-548c-5p downregulates inflammatory response in preeclampsia via targeting PTPRO. J Cell Physiol.

[92] Ma HY, Cu W, Sun YH, Chen X (2020). MiRNA-203a-3p inhibits inflammatory response in preeclampsia through regulating IL24. Eur Rev Med Pharmacol Sci.

[93] Wang Y, Du X, Wang J (2020). Transfer of miR-15a-5p by placental exosomes promotes pre-eclampsia progression by regulating PI3K/AKT signaling pathway via CDK1. Mol Immunol.

[94] Yang X, Meng T (2019). MicroRNA-431 affects trophoblast migration and invasion by targeting ZEB1 in preeclampsia. Gene.

[95] Xueya Z, Yamei L, Sha C, Dan C, Hong S, Xingyu Y, Weiwei C (2020). Exosomal encapsulation of miR-125a-5p inhibited trophoblast cell migration and proliferation by regulating the expression of VEGFA in preeclampsia. Biochem Biophys Res Commun.

[96] Salomon C, Guanzon D, Scholz-Romero K, Longo S, Correa P, Illanes SE, Rice GE (2017). Placental Exosomes as Early Biomarker of Preeclampsia: Potential Role of Exosomal MicroRNAs Across Gestation. J Clin Endocrinol Metab.

[97] Wu Y, Wang Y, Wei M, Han X, Xu T, Cui M (2020). Advances in the study of exosomal lncRNAs in tumors and the selection of research methods. Biomed Pharmacother.

[98] Vento-Tormo R, Efremova M, Botting RA, Turco MY, Vento-Tormo M, Meyer KB, Park JE, Stephenson E, Polański K, Goncalves A, Gardner L, Holmqvist S, Henriksson J, Zou A, Sharkey AM, Millar B, Innes B, Wood L, Wilbrey-Clark A, Payne RP, Ivarsson MA, Lisgo S, Filby A, Rowitch DH, Bulmer JN, Wright GJ, Stubbington MJT, Haniffa M, Moffett A, Teichmann SA (2018). Single-cell reconstruction of the early maternal-fetal interface in humans. Nature.

[99] Guo F, Zhang B, Yang H, Fu Y, Wang Y, Huang J, Cheng M, Li X, Shen Z, Li L, He P, Xiang AP, Wang S, Zhang H (2021). Systemic transcriptome comparison between early- And late-onset pre-eclampsia shows distinct pathology and novel biomarkers. Cell Prolif.

[100] Khong SL, Kane SC, Brennecke SP, da Silva Costa F (2015). First-trimester uterine artery Doppler analysis in the prediction of later pregnancy complications. Dis Markers.

[101] Nicolaides KH (2011). Turning the pyramid of prenatal care. Fetal Diagn Ther.

[102] Magee LA, Pels A, Helewa M, Rey E, von Dadelszen P (2014). Diagnosis, evaluation, and management of the hypertensive disorders of pregnancy. Pregnancy Hypertens.

[103] Bibbins-Domingo K, Grossman DC, Curry SJ, Barry MJ, Davidson KW, Doubeni CA, Epling JW, Kemper AR, Krist AH, Kurth AE, Landefeld CS, Mangione CM, Phillips WR, Phipps MG, Silverstein M, Simon MA, Tseng CW (2017). Screening for Preeclampsia: US Preventive Services Task Force Recommendation Statement. JAMA.

[104] Giorgione V, Ridder A, Kalafat E, Khalil A, Thilaganathan B (2021). Incidence of postpartum hypertension within 2 years of a pregnancy complicated by pre-eclampsia: a systematic review and meta-analysis. BJOG.

[105] Belghiti J, Kayem G, Tsatsaris V, Goffinet F, Sibai BM, Haddad B (2011). Benefits and risks of expectant management of severe preeclampsia at less than 26 weeks gestation: the impact of gestational age and severe fetal growth restriction. Am J Obstet Gynecol.

[106] Wang Y, Hao M, Sampson S, Xia J (2017). Elective delivery versus expectant management for pre-eclampsia: a meta-analysis of RCTs. Arch Gynecol Obstet.

[107] Li X, Han X, Yang J, Bao J, Di X, Zhang G, Liu H (2017). Magnesium Sulfate Provides Neuroprotection in Eclampsia-Like Seizure Model by Ameliorating Neuroinflammation and Brain Edema. Mol Neurobiol.

[108] Sibai BM (2004). Magnesium sulfate prophylaxis in preeclampsia: Lessons learned from recent trials. Am J Obstet Gynecol.

[109] Altman D, Carroli G, Duley L, Farrell B, Moodley J, Neilson J, Smith D (2002). Do women with pre-eclampsia, and their babies, benefit from magnesium sulphate? The Magpie Trial: a randomised placebo-controlled trial. Lancet.

[110] Belfort MA, Anthony J, Saade GR, Allen JC (2003). A comparison of magnesium sulfate and nimodipine for the prevention of eclampsia. N Engl J Med.

[111] Witlin AG, Friedman SA, Sibai BM (1997). The effect of magnesium sulfate therapy on the duration of labor in women with mild preeclampsia at term: a randomized, double-blind, placebo-controlled trial. Am J Obstet Gynecol.

[112] Bohman VR, Cotton DB (1990). Supralethal magnesemia with patient survival. Obstet Gynecol.

[113] Roberts D, Brown J, Medley N, Dalziel SR (2017). Antenatal corticosteroids for accelerating fetal lung maturation for women at risk of preterm birth. Cochrane Database Syst Rev..

[114] Seckl JR, Cleasby M, Nyirenda MJ (2000). Glucocorticoids, 11beta-hydroxysteroid dehydrogenase, and fetal programming. Kidney Int.

[115] Clark PM (1998). Programming of the hypothalamo-pituitary-adrenal axis and the fetal origins of adult disease hypothesis. Eur J Pediatr.

[116] Dodic M, Wintour EM, Whitworth JA, Coghlan JP (1999). Effect of steroid hormones on blood pressure. Clin Exp Pharmacol Physiol.

[117] Edwards LJ, Coulter CL, Symonds ME, McMillen IC (2001). Prenatal undernutrition, glucocorticoids and the programming of adult hypertension. Clin Exp Pharmacol Physiol.

[118] Huang WL, Beazley LD, Quinlivan JA, Evans SF, Newnham JP, Dunlop SA (1999). Effect of corticosteroids on brain growth in fetal sheep. Obstet Gynecol.

[119] Jobe AH, Wada N, Berry LM, Ikegami M, Ervin MG (1998). Single and repetitive maternal glucocorticoid exposures reduce fetal growth in sheep. Am J Obstet Gynecol.

[120] Smith DD, Costantine MM. The role of statins in the prevention of preeclampsia. Am J Obstet Gynecol. 2022;226(2S):S1171–S1181. 10.1016/j.ajog.2020.08.040 PMC8237152 32818477

[121] N Meyer, L Brodowski, K Richter, CS von Kaisenberg, B Schroder-Heurich, F von Versen-Hoynck. Pravastatin Promotes Endothelial Colony-Forming Cell Function, Angiogenic Signaling and Protein Expression In Vitro, J Clin Med. 2021;10(2) 10.3390/jcm10020183 PMC7825508 33419165

[122] Ahmed A, Williams DJ, Cheed V, Middleton LJ, Ahmad S, Wang K, Vince AT, Hewett P, Spencer K, Khan KS, Daniels JP, StAm PTCG (2020). Pravastatin for early-onset pre-eclampsia: a randomised, blinded, placebo-controlled trial. BJOG..

[123] Manson JM, Freyssinges C, Ducrocq MB, Stephenson WP (1996). Postmarketing surveillance of lovastatin and simvastatin exposure during pregnancy. Reprod Toxicol.

[124] Girardi G (2014). Can statins prevent pregnancy complications?. J Reprod Immunol.

[125] Bateman BT, Hernandez-Diaz S, Fischer MA, Seely EW, Ecker JL, Franklin JM, Desai RJ, Allen-Coleman C, Mogun H, Avorn J, Huybrechts KF (2015). Statins and congenital malformations: cohort study. BMJ.

[126] Lecarpentier E, Morel O, Fournier T, Elefant E, Chavatte-Palmer P, Tsatsaris V (2012). Statins and pregnancy: between supposed risks and theoretical benefits. Drugs.

[127] Winterfeld U, Allignol A, Panchaud A, Rothuizen LE, Merlob P, Cuppers-Maarschalkerweerd B, Vial T, Stephens S, Clementi M, De Santis M, Pistelli A, Berlin M, Eleftheriou G, Maňáková E, Buclin T (2013). Pregnancy outcome following maternal exposure to statins: a multicentre prospective study. BJOG.

[128] Kumasawa K, Iriyama T, Nagamatsu T, Osuga Y, Fujii T (2020). Pravastatin for preeclampsia: From animal to human. J Obstet Gynaecol Res.

[129] Brownfoot FC, Hastie R, Hannan NJ, Cannon P, Tuohey L, Parry LJ, Senadheera S, Illanes SE, Kaitu’u-Lino TJ, Tong S (2016). Metformin as a prevention and treatment for preeclampsia: effects on soluble fms-like tyrosine kinase 1 and soluble endoglin secretion and endothelial dysfunction. Am J Obstet Gynecol..

[130] Chiswick C, Reynolds RM, Denison F, Drake AJ, Forbes S, Newby DE, Walker BR, Quenby S, Wray S, Weeks A, Lashen H, Rodriguez A, Murray G, Whyte S, Norman JE (2015). Effect of metformin on maternal and fetal outcomes in obese pregnant women (EMPOWaR): a randomised, double-blind, placebo-controlled trial. Lancet Diabetes Endocrinol.

[131] Feng Y, Yang H (2017). Metformin - a potentially effective drug for gestational diabetes mellitus: a systematic review and meta-analysis. J Matern Fetal Neonatal Med.

[132] Vanky E, Zahlsen K, Spigset O, Carlsen SM (2005). Placental passage of metformin in women with polycystic ovary syndrome. Fertil Steril.

[133] Rowan JA, Rush EC, Obolonkin V, Battin M, Wouldes T, Hague WM (2011). Metformin in gestational diabetes: the offspring follow-up (MiG TOFU): body composition at 2 years of age. Diabetes Care.

[134] Gray SG, McGuire TM, Cohen N, Little PJ (2017). The emerging role of metformin in gestational diabetes mellitus. Diabetes Obes Metab.

[135] Gundelach T, Rodewald M, Bekes I, Janni W, Hancke K (2016). Metformin for the treatment of polycystic ovary syndrome. Med Monatsschr Pharm.

[136] Lautatzis ME, Goulis DG, Vrontakis M (2013). Efficacy and safety of metformin during pregnancy in women with gestational diabetes mellitus or polycystic ovary syndrome: a systematic review. Metabolism.

[137] Meher S, Duley L, Hunter K, Askie L (2017). Antiplatelet therapy before or after 16 weeks' gestation for preventing preeclampsia: an individual participant data meta-analysis. Am J Obstet Gynecol..

[138] Hussain M, Javeed A, Ashraf M, Zhao Y, Mukhtar MM, Rehman MU (2012). Aspirin and immune system. Int Immunopharmacol.

[139] Weber C, Erl W, Pietsch A, Weber PC (1995). Aspirin inhibits nuclear factor-kappa B mobilization and monocyte adhesion in stimulated human endothelial cells. Circulation.

[140] Buckland M, Lombardi G (2009). Aspirin and the induction of tolerance by dendritic cells. Handb Exp Pharmacol..

[141] Javeed A, Zhang B, Qu Y, Zhang A, Sun C, Zhang L, Liu J, Zeng C, Zhao Y (2009). The significantly enhanced frequency of functional CD4+CD25+Foxp3+ T regulatory cells in therapeutic dose aspirin-treated mice. Transpl Immunol.

[142] L Duley, S Meher, KE Hunter, AL Seidler, LM Askie. Antiplatelet agents for preventing pre-eclampsia and its complications. Cochrane Database Syst Rev. 2019;2019(10). 10.1002/14651858.CD004659.pub3 PMC6820858 31684684

[143] Scott G, Gillon TE, Pels A, von Dadelszen P, Magee LA. Guidelines-similarities and dissimilarities: a systematic review of international clinical practice guidelines for pregnancy hypertension. Am J Obstet Gynecol. 2022;226(2S):S1222–S1236. 10.1016/j.ajog.2020.08.018 32828743

[144] Coomarasamy A, Honest H, Papaioannou S, Gee H, Khan KS (2003). Aspirin for prevention of preeclampsia in women with historical risk factors: a systematic review. Obstet Gynecol.

[145] L.M. Askie, L. Duley, D.J. Henderson-Smart, L.A. Stewart, P.C. Group (2007). Antiplatelet agents for prevention of pre-eclampsia: a meta-analysis of individual patient data. Lancet.

[146] Rolnik DL, Nicolaides KH, Poon LC. Prevention of preeclampsia with aspirin. Am J Obstet Gynecol. 2022;226(2S):S1108–S1119. 10.1016/j.ajog.2020.08.045 32835720

[147] Hofmeyr GJ, Lawrie TA, Atallah AN, Torloni MR (2018). Calcium supplementation during pregnancy for preventing hypertensive disorders and related problems. Cochrane Database Syst Rev..

[148] Hofmeyr GJ, Manyame S, Medley N, Williams MJ (2019). Calcium supplementation commencing before or early in pregnancy, for preventing hypertensive disorders of pregnancy. Cochrane Database Syst Rev..

[149] Sun X, Li H, He X, Li M, Yan P, Xun Y, Lu C, Yang K, Zhang X (2019). The association between calcium supplement and preeclampsia and gestational hypertension: a systematic review and meta-analysis of randomized trials. Hypertens Pregnancy.

[150] Hannan NJ, Binder NK, Beard S, Nguyen TV, Kaitu'u-Lino TJ, Tong S (2018). Melatonin enhances antioxidant molecules in the placenta, reduces secretion of soluble fms-like tyrosine kinase 1 (sFLT) from primary trophoblast but does not rescue endothelial dysfunction: An evaluation of its potential to treat preeclampsia. PLoS ONE.

[151] Hobson SR, Gurusinghe S, Lim R, Alers NO, Miller SL, Kingdom JC, Wallace EM (2018). Melatonin improves endothelial function in vitro and prolongs pregnancy in women with early-onset preeclampsia. J Pineal Res.

[152] Suvakov S, Richards C, Nikolic V, Simic T, McGrath K, Krasnodembskaya A, McClements L (2020). Emerging Therapeutic Potential of Mesenchymal Stem/Stromal Cells in Preeclampsia. Curr Hypertens Rep.

[153] Fu L, Liu Y, Zhang D, Xie J, Guan H, Shang T (2015). Beneficial effect of human umbilical cord-derived mesenchymal stem cells on an endotoxin-induced rat model of preeclampsia. Exp Ther Med.

[154] Grimes S, Bombay K, Lanes A, Walker M, Corsi DJ (2019). Potential biological therapies for severe preeclampsia: a systematic review and meta-analysis. BMC Pregnancy Childbirth.

[155] Hahn S (2015). Preeclampsia - will orphan drug status facilitate innovative biological therapies?. Front Surg.

[156] Charif N, Li YY, Targa L, Zhang L, Ye JS, Li YP, Stoltz JF, Han HZ, de Isla N (2017). Aging of bone marrow mesenchymal stromal/stem cells: Implications on autologous regenerative medicine. Biomed Mater Eng.

[157] T Tilburgs, C Crespo Â, A van der Zwan, B Rybalov, T Raj, B Stranger, L Gardner, A Moffett, JL Strominger. Human HLA-G+ extravillous trophoblasts: Immune-activating cells that interact with decidual leukocytes. Proc Natl Acad Sci U S A. 2015;112(23):7219–24. 10.1073/pnas.1507977112 PMC4466754 26015573

[158] Liu L, Zhao G, Fan H, Zhao X, Li P, Wang Z, Hu Y, Hou Y (2014). Mesenchymal stem cells ameliorate Th1-induced pre-eclampsia-like symptoms in mice via the suppression of TNF-alpha expression. PLoS ONE.

[159] Wang L, Xu X, Kang L, Xiang W (2016). Bone Marrow Mesenchymal Stem Cells Attenuate Mitochondria Damage Induced by Hypoxia in Mouse Trophoblasts. PLoS ONE.

[160] Prather WR, Toren A, Meiron M (2008). Placental-derived and expanded mesenchymal stromal cells (PLX-I) to enhance the engraftment of hematopoietic stem cells derived from umbilical cord blood. Expert Opin Biol Ther.

[161] Prather WR, Toren A, Meiron M, Ofir R, Tschope C, Horwitz EM (2009). The role of placental-derived adherent stromal cell (PLX-PAD) in the treatment of critical limb ischemia. Cytotherapy.

[162] Lahiani A, Zahavi E, Netzer N, Ofir R, Pinzur L, Raveh S, Arien-Zakay H, Yavin E, Lazarovici P (2015). Human placental eXpanded (PLX) mesenchymal-like adherent stromal cells confer neuroprotection to nerve growth factor (NGF)-differentiated PC12 cells exposed to ischemia by secretion of IL-6 and VEGF. Biochim Biophys Acta.

[163] Todd N, McNally R, Alqudah A, Jerotic D, Suvakov S, Obradovic D, Hoch D, Hombrebueno JR, Campos GL, Watson CJ, Gojnic-Dugalic M, Simic TP, Krasnodembskaya A, Desoye G, Eastwood KA, Hunter AJ, Holmes VA, McCance DR, Young IS, Grieve DJ, Kenny LC, Garovic VD, Robson T, McClements L (2021). Role of A Novel Angiogenesis FKBPL-CD44 Pathway in Preeclampsia Risk Stratification and Mesenchymal Stem Cell Treatment. J Clin Endocrinol Metab.

[164] Annett S, Moore G, Short A, Marshall A, McCrudden C, Yakkundi A, Das S, McCluggage WG, Nelson L, Moustafa N, Kennedy CJ, deFazio A, Brand A, Sharma R, Brennan D, O'Toole S, O'Leary J, Bates M, O'Riain C, O'Connor D, Furlong F, McCarthy H, Kissenpfennig A, McClements L, Robson T (2020). FKBPL-based peptide, ALM201, targets angiogenesis and cancer stem cells in ovarian cancer. Br J Cancer.

[165] Zhang Z, Wang X, Wang J, Zhang L (2018). The decreased expression of Stat3 and p-Stat3 in preeclampsia-like rat placenta. J Mol Histol.

[166] Chen X, Tong C, Li H, Peng W, Li R, Luo X, Ge H, Ran Y, Li Q, Liu Y, Xiong X, Bai Y, Zhang H, Baker PN, Liu X, Qi H (2018). Dysregulated Expression of RPS4Y1 (Ribosomal Protein S4, Y-Linked 1) Impairs STAT3 (Signal Transducer and Activator of Transcription 3) Signaling to Suppress Trophoblast Cell Migration and Invasion in Preeclampsia. Hypertension.

[167] Gupta M, Brewer G (2006). MicroRNAs: new players in an old game. Proc Natl Acad Sci U S A.

[168] Kim VN, Nam JW (2006). Genomics of microRNA. Trends Genet.

[169] Avissar M, Christensen BC, Kelsey KT, Marsit CJ (2009). MicroRNA expression ratio is predictive of head and neck squamous cell carcinoma. Clin Cancer Res.

[170] Yu Z, Willmarth NE, Zhou J, Katiyar S, Wang M, Liu Y, McCue PA, Quong AA, Lisanti MP, Pestell RG (2010). microRNA 17/20 inhibits cellular invasion and tumor metastasis in breast cancer by heterotypic signaling. Proc Natl Acad Sci U S A.

[171] Sun L, Li D, Song K, Wei J, Yao S, Li Z, Su X, Ju X, Chao L, Deng X, Kong B, Li L (2017). Exosomes derived from human umbilical cord mesenchymal stem cells protect against cisplatin-induced ovarian granulosa cell stress and apoptosis in vitro. Sci Rep.

[172] Hu TX, Guo X, Wang G, Gao L, He P, Xia Y, Gu H, Ni X (2017). MiR133b is involved in endogenous hydrogen sulfide suppression of sFlt-1 production in human placenta. Placenta.

[173] Kong C, Sun L, Zhang M, Ding L, Zhang Q, Cheng X, Diao Z, Yan Q, Zhang H, Fang T, Zhen X, Hu Y, Sun H, Yan G (2016). miR-133b Reverses the Hydrosalpinx-induced Impairment of Embryo Attachment Through Down-regulation of SGK1. J Clin Endocrinol Metab.

[174] Wang D, Na Q, Song GY, Wang L (2020). Human umbilical cord mesenchymal stem cell-derived exosome-mediated transfer of microRNA-133b boosts trophoblast cell proliferation, migration and invasion in preeclampsia by restricting SGK1. Cell Cycle.

[175] Pineles BL, Romero R, Montenegro D, Tarca AL, Han YM, Kim YM, Draghici S, Espinoza J, Kusanovic JP, Mittal P, Hassan SS, Kim CJ (2007). Distinct subsets of microRNAs are expressed differentially in the human placentas of patients with preeclampsia. Am J Obstet Gynecol.

[176] Zhu XM, Han T, Sargent IL, Yin GW, Yao YQ (2009). Differential expression profile of microRNAs in human placentas from preeclamptic pregnancies vs normal pregnancies. Am J Obstet Gynecol.

[177] Lee DC, Romero R, Kim JS, Tarca AL, Montenegro D, Pineles BL, Kim E, Lee J, Kim SY, Draghici S, Mittal P, Kusanovic JP, Chaiworapongsa T, Hassan SS, Kim CJ (2011). miR-210 targets iron-sulfur cluster scaffold homologue in human trophoblast cell lines: siderosis of interstitial trophoblasts as a novel pathology of preterm preeclampsia and small-for-gestational-age pregnancies. Am J Pathol.

[178] Zhang D, Fu L, Wang L, Lin L, Yu L, Zhang L, Shang T (2017). Therapeutic benefit of mesenchymal stem cells in pregnant rats with angiotensin receptor agonistic autoantibody-induced hypertension: Implications for immunomodulation and cytoprotection. Hypertens Pregnancy.

[179] Vakhshiteh F, Atyabi F, Ostad SN (2019). Mesenchymal stem cell exosomes: a two-edged sword in cancer therapy. Int J Nanomedicine.

[180] de Alwis N, Binder NK, Beard S, Kaitu'u-Lino TJ, Tong S, Brownfoot F, Hannan NJ (2020). Novel approaches to combat preeclampsia: from new drugs to innovative delivery. Placenta.

[181] Kim BY, Rutka JT, Chan WC (2010). Nanomedicine. N Engl J Med.

[182] Bamrungsap S, Zhao Z, Chen T, Wang L, Li C, Fu T, Tan W (2012). Nanotechnology in therapeutics: a focus on nanoparticles as a drug delivery system. Nanomedicine (Lond).

[183] MacDiarmid JA, Mugridge NB, Weiss JC, Phillips L, Burn AL, Paulin RP, Haasdyk JE, Dickson KA, Brahmbhatt VN, Pattison ST, James AC, Al Bakri G, Straw RC, Stillman B, Graham RM, Brahmbhatt H (2007). Bacterially derived 400 nm particles for encapsulation and cancer cell targeting of chemotherapeutics. Cancer Cell..

[184] Keelan JA, Leong JW, Ho D, Iyer KS (2015). Therapeutic and safety considerations of nanoparticle-mediated drug delivery in pregnancy. Nanomedicine (Lond).

[185] Lian T, Ho RJ (2001). Trends and developments in liposome drug delivery systems. J Pharm Sci.

[186] Lee CC, MacKay JA, Fréchet JM, Szoka FC (2005). Designing dendrimers for biological applications. Nat Biotechnol.

[187] Connor EE, Mwamuka J, Gole A, Murphy CJ, Wyatt MD (2005). Gold nanoparticles are taken up by human cells but do not cause acute cytotoxicity. Small.

[188] Kaitu'u-Lino TJ, Pattison S, Ye L, Tuohey L, Sluka P, MacDiarmid J, Brahmbhatt H, Johns T, Horne AW, Brown J, Tong S (2013). Targeted nanoparticle delivery of doxorubicin into placental tissues to treat ectopic pregnancies. Endocrinology.

[189] Jingting C, Huining L, Yi Z (2011). Preparation and characterization of magnetic nanoparticles containing Fe(3)O(4)-dextran-anti-β-human chorionic gonadotropin, a new generation choriocarcinoma-specific gene vector. Int J Nanomedicine.

[190] Crews JK, Herrington JN, Granger JP, Khalil RA (2000). Decreased endothelium-dependent vascular relaxation during reduction of uterine perfusion pressure in pregnant rat. Hypertension.

[191] Kumasawa K, Ikawa M, Kidoya H, Hasuwa H, Saito-Fujita T, Morioka Y, Takakura N, Kimura T, Okabe M (2011). Pravastatin induces placental growth factor (PGF) and ameliorates preeclampsia in a mouse model. Proc Natl Acad Sci U S A.

[192] Davisson RL, Hoffmann DS, Butz GM, Aldape G, Schlager G, Merrill DC, Sethi S, Weiss RM, Bates JN (2002). Discovery of a spontaneous genetic mouse model of preeclampsia. Hypertension.

[193] Kanasaki K, Palmsten K, Sugimoto H, Ahmad S, Hamano Y, Xie L, Parry S, Augustin HG, Gattone VH, Folkman J, Strauss JF, Kalluri R (2008). Deficiency in catechol-O-methyltransferase and 2-methoxyoestradiol is associated with pre-eclampsia. Nature.

[194] Takimoto E, Ishida J, Sugiyama F, Horiguchi H, Murakami K, Fukamizu A (1996). Hypertension induced in pregnant mice by placental renin and maternal angiotensinogen. Science.

[195] Zhang J, Chen Z, Smith GN, Croy BA (2011). Natural killer cell-triggered vascular transformation: maternal care before birth?. Cell Mol Immunol.

[196] Romero R, Nien JK, Espinoza J, Todem D, Fu W, Chung H, Kusanovic JP, Gotsch F, Erez O, Mazaki-Tovi S, Gomez R, Edwin S, Chaiworapongsa T, Levine RJ, Karumanchi SA (2008). A longitudinal study of angiogenic (placental growth factor) and anti-angiogenic (soluble endoglin and soluble vascular endothelial growth factor receptor-1) factors in normal pregnancy and patients destined to develop preeclampsia and deliver a small for gestational age neonate. J Matern Fetal Neonatal Med.

[197] Rana S, Powe CE, Salahuddin S, Verlohren S, Perschel FH, Levine RJ, Lim KH, Wenger JB, Thadhani R, Karumanchi SA (2012). Angiogenic factors and the risk of adverse outcomes in women with suspected preeclampsia. Circulation.

[198] Poon LC, Kametas NA, Maiz N, Akolekar R, Nicolaides KH (2009). First-trimester prediction of hypertensive disorders in pregnancy. Hypertension.

[199] Muzzio D, Foglia ML, Desimone MF, Zygmunt M (2017). 3D In Vitro Models of Early Pregnancy: How to Choose the Right Scaffolding Material?. Curr Pharm Des.

[200] Haider S, Meinhardt G, Saleh L, Kunihs V, Gamperl M, Kaindl U, Ellinger A, Burkard TR, Fiala C, Pollheimer J, Mendjan S, Latos PA, Knöfler M (2018). Self-Renewing Trophoblast Organoids Recapitulate the Developmental Program of the Early Human Placenta. Stem Cell Reports.

